# Cellular senescence in cancer: from mechanism paradoxes to precision therapeutics

**DOI:** 10.1186/s12943-025-02419-2

**Published:** 2025-08-08

**Authors:** Tiejun Feng, Fuda Xie, Leo M.Y. Lee, Zhiqiang Lin, Yifan Tu, Yang Lyu, Peiyao Yu, Jialin Wu, Bonan Chen, Ge Zhang, Gary M.K. Tse, Ka Fai To, Wei Kang

**Affiliations:** 1 Department of Anatomical and Cellular Pathology, State Key Laboratory of Translational Oncology, Prince of Wales Hospital, Sir Y.K. Pao Cancer Center, The Chinese University of Hong Kong, Hong Kong, China; 2 Institute of Digestive Disease, State Key Laboratory of Digestive Disease, Li Ka Shing Institute of Health Science, The Chinese University of Hong Kong, Hong Kong, China; 3 CUHK-Shenzhen Research Institute, Shenzhen, China; 4 Department of Applied Biology and Chemical Technology, The Hong Kong Polytechnic University, Hong Kong, China; 5 National Clinical Research Center for Infectious Disease, Shenzhen Third People’s Hospital, Southern University of Science and Technology, Shenzhen, China; 6 Department of Obstetrics and Gynaecology, The Chinese University of Hong Kong, Hong Kong, China; 7 Law Sau Fai Institute for Advancing Translational Medicine in Bone and Joint Diseases (TMBJ), School of Chinese Medicine, Hong Kong Baptist University, Hong Kong, China

**Keywords:** Senescence, Cancer, Therapeutic innovation

## Abstract

Cellular senescence is a double-edged sword in cancer biology, functioning as both a tumor-suppressive mechanism and a driver of malignancy. Initially, senescence acts as a protective barrier by arresting the proliferation of damaged or oncogene-expressing cells via pathways such as oncogene-induced senescence and the DNA damage response. However, persistent senescence-associated secretory phenotype and metabolic reprogramming in senescent cells create a pro-inflammatory, immunosuppressive tumor microenvironment, fueling cancer progression, therapy resistance, and metastasis. This comprehensive review systematically examines the molecular mechanisms of senescence across diverse cancers, spanning digestive, reproductive, urinary, respiratory, nervous, hematologic, endocrine, and integumentary systems, and elucidates its context-dependent roles in tumor suppression and promotion. We highlight groundbreaking therapeutic innovations, including precision senolytics, senomorphics, and combinatorial strategies integrating immunotherapy, metabolic interventions, and epigenetic modulators. The review also addresses microenvironment remodeling and cutting-edge technologies for dissecting senescence heterogeneity, epigenetic clocks for biological age prediction, and microbiome engineering to modulate senescence. Despite their promise, challenges such as off-target effects, biomarker limitations, and cellular heterogeneity underscore the need for precision medicine approaches. Finally, we propose future directions to harness senescence as a dynamic therapeutic target, offering transformative potential for cancer treatment.

## Introduction

Cancer and aging are two complex biological processes that share deep molecular connections. While aging is characterized by a progressive decline in physiological function, cancer arises from uncontrolled cellular proliferation. Despite their seemingly opposing nature, with aging characterized by reduced cell division and cancer defined by uncontrolled growth, these processes are fundamentally interconnected. They share common underlying mechanisms including genomic instability, metabolic reprogramming, epigenetic alterations, and chronic inflammation [1].

The risk of most cancers increases exponentially with age, with over 60% of malignancies diagnosed in individuals aged 65 and older [2]. This association is particularly evident in solid tumors, including lung, breast, prostate, and gastrointestinal cancers. The accumulation of DNA damage, chronic inflammation, and declining immune surveillance (immunosenescence) create a permissive environment for malignant transformation [3]. Additionally, cellular senescence, a state of irreversible growth arrest, plays a dual role in cancer: it acts as a tumor suppressive mechanism early in life but contributes to chronic inflammation and tissue dysfunction in later years, paradoxically promoting cancer progression [4].

Chronic low-grade inflammation, termed “inflammaging”, further links aging and cancer. Elevated levels of pro-inflammatory cytokines such as IL6 and TNF-α, along with senescent cell accumulation, foster a tumor-permissive microenvironment [5, 6]. Metabolic dysregulation, including mitochondrial dysfunction and altered nutrient sensing pathways such as mTOR and AMPK, also influences both aging and oncogenesis [7, 8]. Notably, many longevity-associated pathways, such as insulin/IGF1 signaling and sirtuin activity, are frequently dysregulated in cancer [9].

Therapies targeting aging-related pathways, such as senolytics, senomorphics, immunotherapy, metabolic interventions, epigenetic modulators, and microenvironment remodeling drugs, are now being explored for cancer prevention and treatment. However, challenges remain, including the need for personalized approaches that account for biological (rather than chronological) age and the potential side effects of anti-aging interventions in cancer patients [10].

Understanding the bidirectional relationship between aging and cancer is crucial for developing strategies to delay cancer onset, improve treatment tolerance in elderly patients, and enhance long-term survival. This review explores the dual role of senescence, the relation between senescence and specific cancer, and emerging therapeutic opportunities at this critical intersection.

## Mechanisms of senescence in cancers

### Senescence as a tumor suppressor

#### Oncogene-induced senescence in tumors

Oncogene-induced senescence (OIS) is a critical tumor suppressive mechanism that acts as an initial barrier to cancer development. It is characterized by a stable cell-cycle arrest in response to oncogenic signals, which prevents the proliferation of potentially malignant cells.

**Fig. 1:**
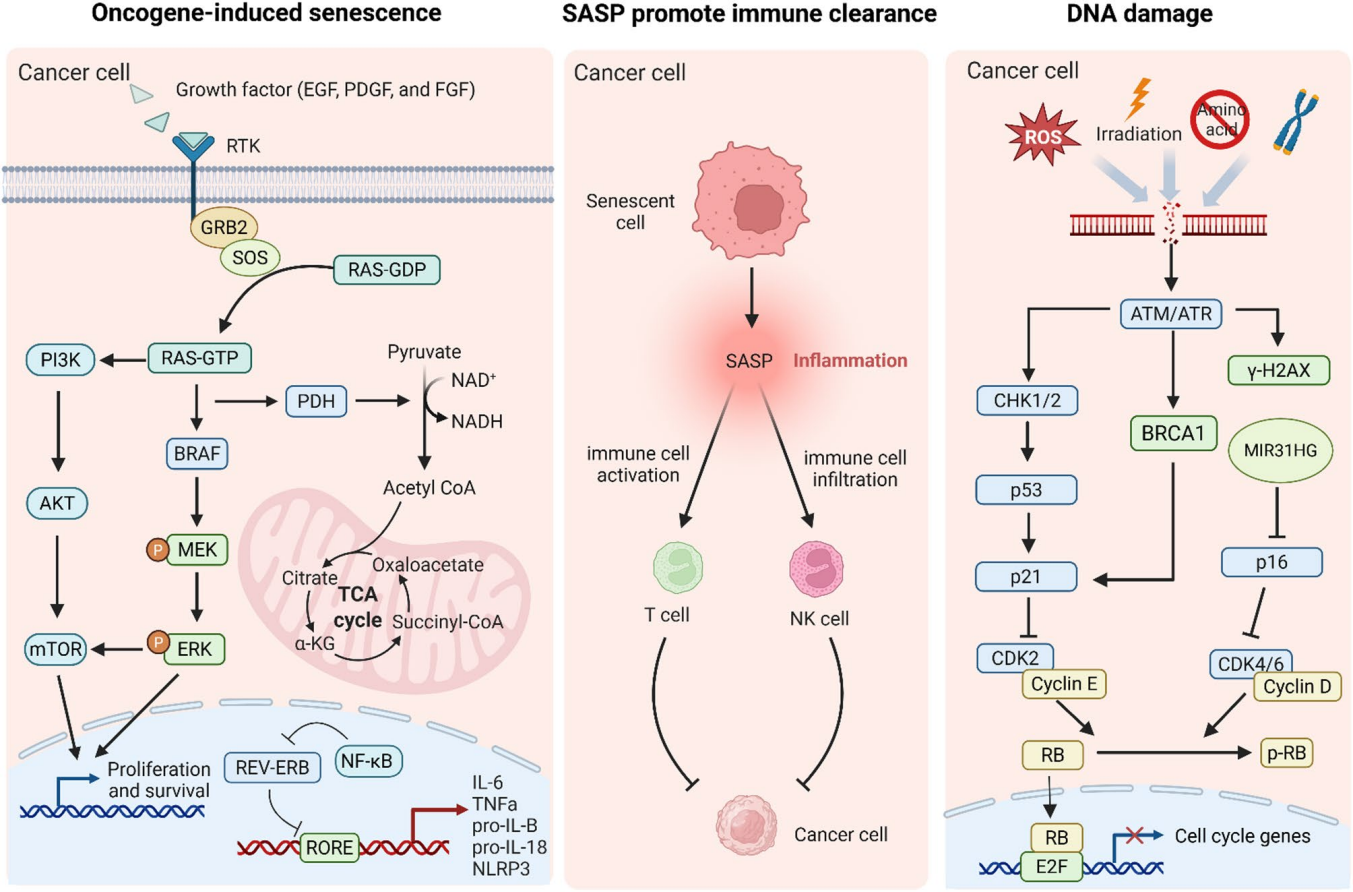
Molecular pathways of oncogene-induced senescence, SASP promote immune clearance, and DNA damage. Oncogenic signaling activation: Growth factors (EGF, PDGF, FGF) bind RTKs, activating RAS-GTP and downstream pathways including PI3K-AKT-mTOR and BRAF-MEK-ERK to drive proliferation. Metabolic changes (NAD+/NADH, TCA cycle) contribute to senescence establishment. SASP-mediated immune clearance: Senescent cells secrete SASP factors that recruit and activate immune cells (T cells, NK cells) for tumor cell elimination. DNA damage: DNA damage activates ATM/ATR-CHK1/2-p53-p21-RB and p16-RB pathways, leading to cell cycle arrest. *Abbreviations*: *EGF* Epidermal Growth Factor, *PDGF* Platelet-Derived Growth Factor, *FGF* Fibroblast Growth Factor, *RTK* Receptor Tyrosine Kinase, *GRB2* Growth Factor Receptor-Bound Protein 2, *SOS* Son of Sevenless, *RAS-GDP* RAS Guanosine Diphosphate, *RAS-GTP* RAS Guanosine Triphosphate, *PDH* Pyruvate Dehydrogenase, *MEK* Mitogen-Activated Protein Kinase Kinase, *ERK* Extracellular Signal-Regulated Kinase, *REV-ERB* Reverse ErbA-related orphan receptor, *RORE* Retinoic Acid Receptor-Related Orphan Receptor Response Element, *SASP* Senescence-Associated Secretory Phenotype, *ATM/ATR* Ataxia Telangiectasia Mutated/ATM and Rad3-Related, *CHK1/2* Checkpoint Kinase 1/2, *CDK2* Cyclin-Dependent Kinase 2, *RB* Retinoblastoma Protein, *E2F* E2F Transcription Factor

One of the key roles of OIS is to prevent the progression of benign lesions into malignant tumors. For instance, in human melanocytic nevi, which are benign lesions harboring activated oncogenes such as BRAF, OIS acts as a barrier to prevent further progression to melanoma (Fig. 1**)**. The growth factor such as EGF, PDGF, and FGF binds to a receptor tyrosine kinase, inducing dimerization and autophosphorylation of its intracellular domain [11]. This recruits the adaptor protein GRB2 and the guanine nucleotide exchange factor SOS, which activates RAS by converting it from its inactive RAS-GDP form to active RAS-GTP [12]. Activated RAS then triggers the MAPK cascade by binding and activating the kinase BRAF (an RAF family member). BRAF phosphorylates MEK, which subsequently phosphorylates and activates ERK, a key mitogen-activated protein kinase. Once activated, ERK translocates to the nucleus, where it phosphorylates transcription factors such as c-Fos and c-Myc, upregulating genes that promote cell cycle progression, survival, and proliferation [13]. Targeted therapies such as BRAF inhibitors (vemurafenib) and MEK inhibitors (trametinib) are used to block hyperactive signaling in tumors. The disruption of OIS may suppress the PI3K pathway through PI3K and mTOR, potentially attenuating tumor progression [14, 15]. This underscores the critical role of OIS in preserving cellular homeostasis and preventing cancer development [16, 17]. The molecular mechanisms underlying OIS involve various regulatory proteins. For example, the mitochondrial gatekeeper pyruvate dehydrogenase (PDH) has been identified as a crucial mediator of OIS induced by the BRAF(V600E) oncogene. The activation of PDH enhances pyruvate utilization in the tricarboxylic acid cycle, leading to increased respiration and redox stress, which are essential for the induction of senescence [18]. Furthermore, the role of the circadian clock in regulating OIS has been explored, with nuclear hormone receptors REVERB-α and REVERB-β being identified as key components. REVERB, a circadian nuclear receptor, binds to retinoic acid receptor-related orphan receptor response elements and recruits corepressors such as NCoR and HDAC3 to suppress pro-inflammatory genes [19]. NF-κB further amplifies inflammation by antagonizing REVERB repression [20]. Pharmacological activation of REVERB receptors has been shown to be lethal to cancer cells and OIS cells, suggesting a potential therapeutic strategy for targeting cancer through the modulation of circadian regulators [21]. Interestingly, the interplay between OIS and the immune system is also significant. Oncogene-expressing senescent melanocytes have been found to upregulate MHC class II, which may promote tumor suppression by activating the adaptive immune system. This suggests that OIS not only acts through cell-autonomous mechanisms but also involves noncell-autonomous effects that can influence the tumor microenvironment [22]. Overall, OIS serves as a crucial tumor suppressive mechanism by halting the proliferation of cells with oncogenic mutations and engaging the immune system to clear these cells.

#### Short senescence-associated secretory phenotype promotes immune clearance

The senescence-associated secretory phenotype (SASP) is a complex mixture of cytokines, chemokines, growth factors, and proteases secreted by senescent cells. While chronic SASP can foster tumor progression and immunosuppression, emerging evidence suggests that acute or short-term SASP exposure may trigger immune-mediated clearance of senescent cells, acting as a tumor suppressive mechanism. This duality underscores the context-dependent role of SASP in cancer immunity.

Short-term SASP secretion recruits and activates immune cells, particularly NK cells and T cells, to eliminate senescent cells. For example: In ER + breast cancer (BC), Kv11.1 channel-induced senescence triggered SASP-dependent activation of CD4 + Th1 cells and memory T cells, leading to TNF-α-mediated killing of senescent tumor cells [23]. In rectal cancer, post-neoadjuvant therapy SASP factors such as IL6, IL8, and CCL5 were correlated with increased CD8 + T-cell infiltration and improved tumor regression [24]. Key SASP factors act as chemoattractants and immune modulators. IL6 and IL8 recruit cytotoxic T cells and NK cells, as observed in pituitary tumors [25]. CCL5 and CXCL1 enhance immune cell trafficking, as seen in acute ischemic stroke models [26]. Some senescent cells, particularly in early-stage tumors, secrete factors that recruit immune cells or directly inhibit neighboring cancer cells. For example, bladder uroepithelial senescent cells maintain tissue integrity by reinforcing the urine-blood barrier [27]. In GC, some senescent stromal cells may exert immunomodulatory effects that enhance immunotherapy efficacy [28]. In KRAS-mutant lung cancer, MAPK and CDK4/6 inhibitors induce senescence and SASP activation, leading to NK cell-mediated tumor cell death through SASP factors such as TNF-α and ICAM-1 [29]. Similarly, in pancreatic ductal adenocarcinoma (PDAC), therapy-induced senescence triggers a pro-angiogenic SASP that enhances CD8 + T cell infiltration, sensitizing tumors to PD-1 blockade [30]. Collectively, these studies underscore the multifaceted role of SASP in immune clearance. Short-lived SASP creates a pro-inflammatory “hot” microenvironment that sustains immune activity and acts as a transient “danger signal” to recruit immune effectors, highlighting its potential as a therapeutic lever. Balancing SASP duration and composition is critical to avoid tipping toward pro-tumorigenic chronic inflammation.

#### DNA damage response activates senescence to prevent malignant transformation

The DNA Damage Response (DDR) is a critical safeguard mechanism that detects and repairs genomic lesions, preventing the accumulation of mutations that drive malignant transformation. When damage is irreparable, DDR triggers cellular senescence, a stable cell-cycle arrest that acts as a tumor-suppressive barrier. This process is mediated by key regulators such as p53, p16, and ATR. ATM/ATR kinases are activated by DNA breaks or replication stress, phosphorylating CHK1/2, which in turn stabilizes p53, inducing senescence by upregulating CDK inhibitor p21 [31]. p21 halts cell cycle progression by inhibiting Cyclin-CDK complexes. This prevents RB phosphorylation, keeping RB bound to E2F and blocking cell cycle gene expression [32]. In lung cancer, lactate dehydrogenase B (LDHB) silencing exacerbated DDR (γ-H2AX foci) and triggered p53-dependent G1/S or G2/M arrest, reinforcing senescence as a failsafe against genomic instability [33]. In kidney injury models, BRCA1 deletion reduced senescence markers (p16 INK4a, SA-β-gal) and fibrosis, revealing its role in promoting senescence to limit tissue damage [34]. Mechanistically, BRCA1 may be regulated by ATM/ATR to facilitate p21 activation, further linking it to senescence control [35, 36]. In addition to these pathways, the role of the tumor suppressor p16 INK4a in OIS is well-established. The lncRNA MIR31HG has been shown to regulate p16 INK4a expression, thereby modulating the senescence response. This regulatory mechanism highlights the intricate network of gene expression control involved in OIS and its potential implications for cancer therapy [37].

The derivative 4-octyl itaconate induced senescence in melanoma by depleting glutathione, elevating ROS, and activating DDR markers (γ-H2AX, β-galactosidase) [38]. Critically short telomeres activate DDR-driven senescence, preventing malignant proliferation through p53/p21-dependent arresting [39, 40]. In prostate cancer (PCa), ALDH1A3 knockdown enhanced radiation-induced senescence while suppressing pro-tumorigenic SASP via cGAS-STING inhibition [41]. The primate-specific isoform RBM38c promoted DDR-induced autophagy to mitigate apoptosis, suggesting dual targeting of DDR and autophagy could optimize senolytic strategies [42]. DDR-activated senescence is a pivotal anti-cancer mechanism, but its context-dependent outcomes, such as SASP-mediated inflammation, necessitate precise therapeutic modulation. Targeting DDR components such as ATR, BRCA1, or telomeres could enhance senescence-based therapies while minimizing resistance.

### Senescence as a tumor promoter

#### Chronic SASP-driven malignancy

SASP plays a significant role in the progression and malignancy of various cancers. SASP can influence cancer progression through several mechanisms, including enhancing cancer cell proliferation, invasion, metastasis, and therapy resistance.

**Fig. 2:**
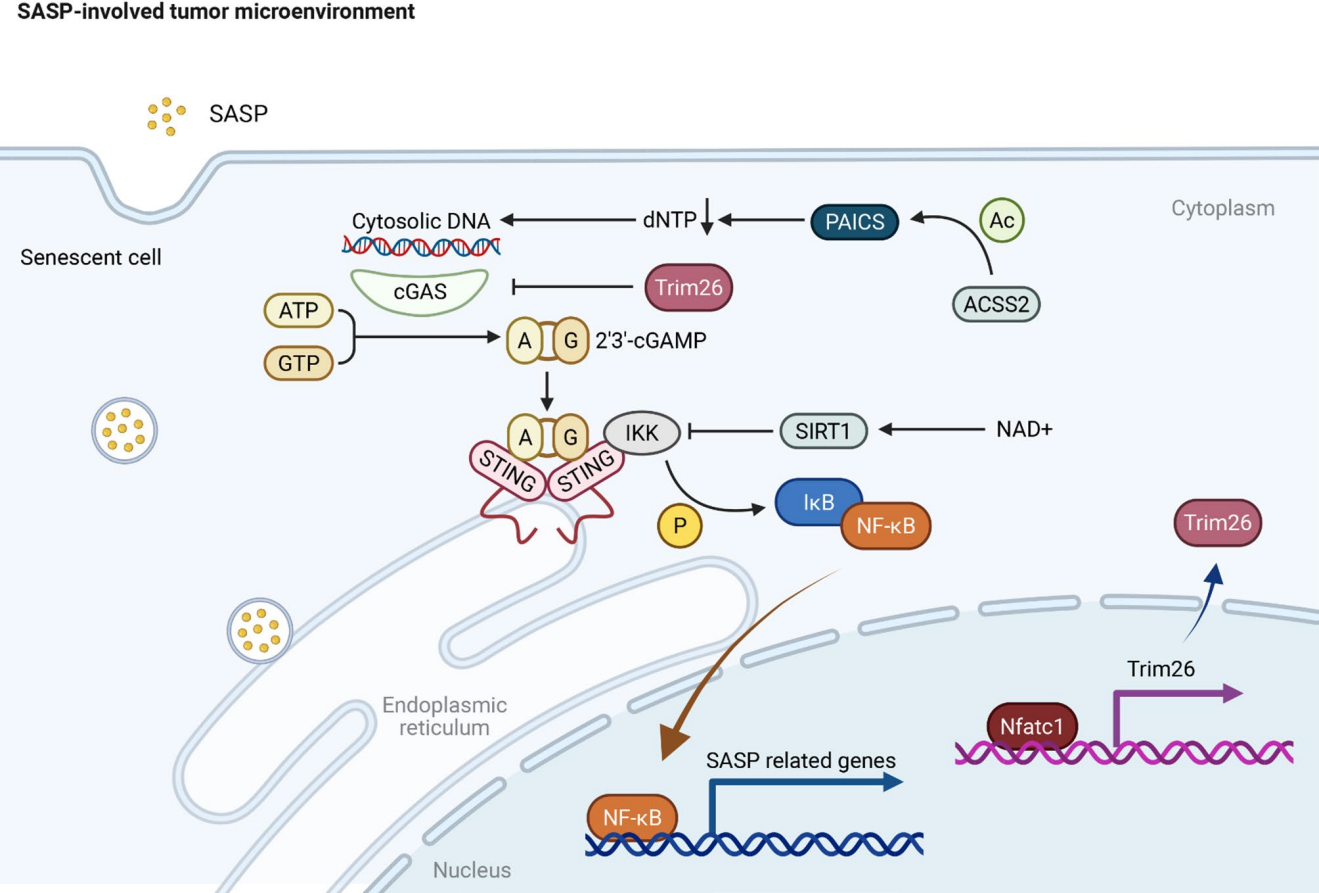
SASP in the tumor microenvironment. Senescent cells accumulate cytosolic DNA, which is detected by the DNA sensor cGAS, leading to the synthesis of the second messenger 2’3’-cGAMP. This activates the STING pathway, triggering downstream IKK signaling, ultimately promoting the nuclear translocation of NF-κB. NF-κB drives the expression of SASP-related genes, which encode pro-inflammatory cytokines, chemokines, and growth factors. The metabolic enzyme ACSS2 and the NAD+-dependent deacetylase SIRT1 modulate this process, while Trim26 regulates both NF-κB and cGAS-STING signaling. Additionally, PAICS, involved in de novo purine synthesis, contributes to the availability of ATP and GTP, further supporting SASP activation. *Abbreviations*: *PAICS* Phosphoribosylaminoimidazole Carboxylase and Succinyltransferase, *AC* Acetyl-CoA, *cGAS* Cyclic GMP-AMP Synthase, *STING* Stimulator of Interferon Genes

In Fig. 2, one of the key pathways involved in SASP-driven malignancy is the cGAS-STING pathway, which is activated by cytosolic DNA fragments from senescent fibroblasts. This pathway is a major regulator of the SASP and has been shown to drive inflammation and tumor progression in aged tissues. The inhibition of this pathway has been suggested as a potential therapeutic strategy to reduce SASP-induced inflammation and improve health-span [43]. In PDAC, cancer-associated fibroblasts (CAFs) exhibiting SASP characteristics have been shown to suppress tumor immunity and promote tumor growth. These fibroblasts can directly suppress the function of tumor-infiltrating CD8 + T cells, thereby limiting the effectiveness of immune checkpoint blockade therapies [44]. This highlights the role of SASP in creating an immunosuppressive tumor microenvironment that supports cancer progression. The ACSS2-PAICS axis was found to regulate SASP by modulating purine metabolism in human primary embryonic lung fibroblast. Pharmacological inhibition of ACSS2 attenuated SASP production and enhanced immune surveillance in hepatic tissue [45]. In triple-negative breast cancer (TNBC), melatonin deficiency exacerbated macrophage senescence via the cGAS-STING pathway, impairing immune clearance. Conversely, melatonin restored Trim26-mediated suppression of SASP by inhibiting the activation of the cGAS-STING pathway, enhancing chemotherapy response in macrophages [46]. The role of SASP in therapy resistance is also significant. Senescent cells can remain metabolically active and secrete SASP factors that contribute to therapy resistance by promoting epithelial-to-mesenchymal transition, invasion, and metastasis. This has been observed in various cancer types, where therapy-induced senescence (TIS) can lead to a paradoxical increase in tumor aggressiveness due to SASP [47]. In glioma, the CCNB2 has been identified as a key driver of malignant transformation. This protein induces SASP in glioma cells, promoting invasion and excessive proliferation through the secretion of SASP cytokines [48]. This demonstrates the direct impact of SASP on the malignant progression of tumors. Furthermore, SASP can influence the genomic landscape of cancer. For instance, in lung adenocarcinoma, genomic rearrangements and mutations can be driven by endogenous processes influenced by SASP, leading to oncogene activation and cancer progression [49]. This suggests that SASP not only affects the tumor microenvironment but also contributes to the genetic evolution of cancer cells. Overall, the SASP represents a complex mechanism that can drive cancer progression and malignancy through multiple pathways.

#### Metabolic reprogramming-driven malignancy and nutrient competition

**Fig. 3:**
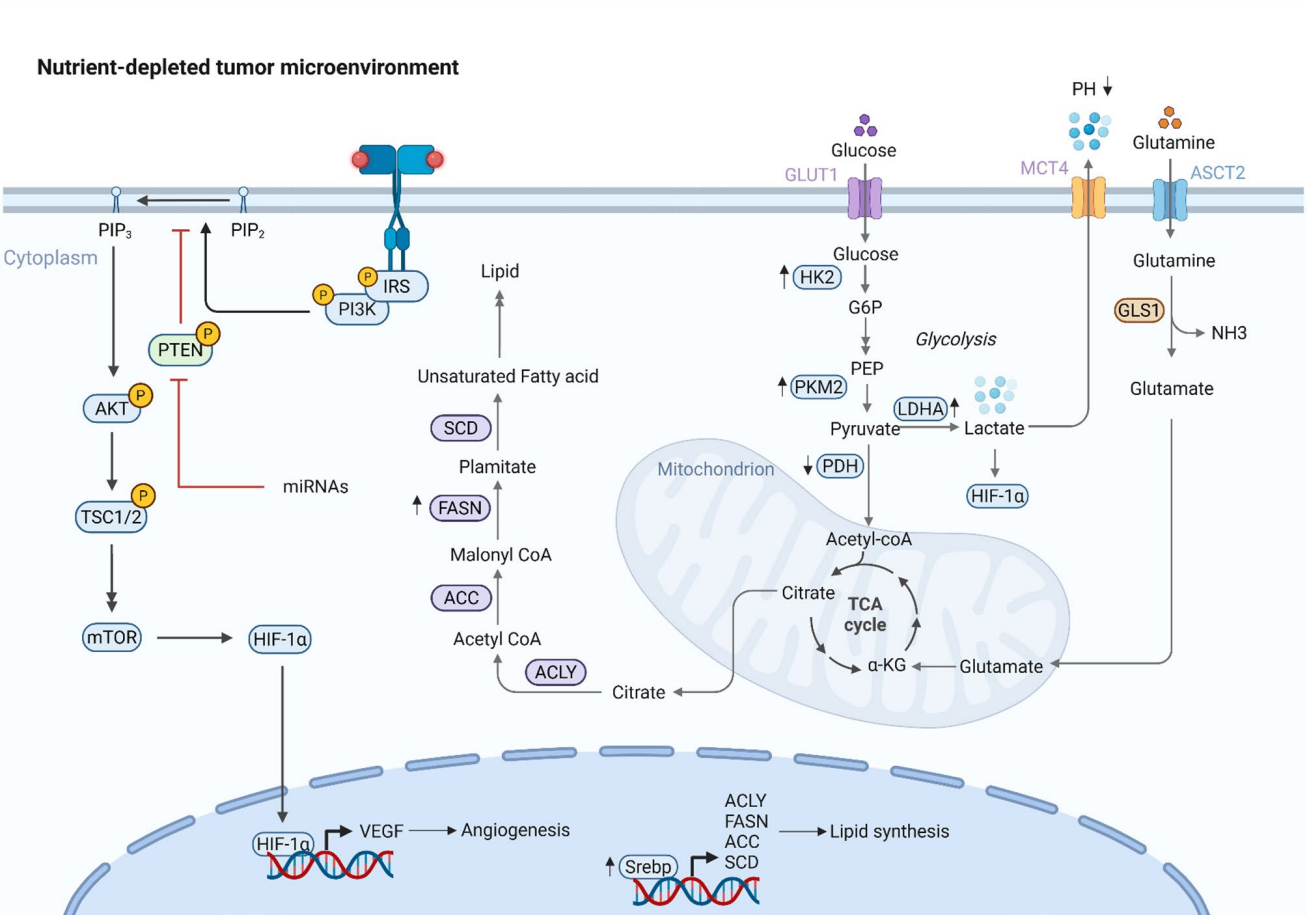
Metabolic reprogramming in cancer cells. Glucose is taken up via GLUT1 and rapidly metabolized through glycolysis, with pyruvate converted to lactate by LDHA, even under aerobic conditions. Mitochondrial metabolism remains active, with pyruvate entering via PDH to generate acetyl-CoA for the TCA cycle, while glutamine is processed by GLS1 into α-KG to sustain energy and biosynthetic precursors. Citrate from the TCA cycle is exported to the cytoplasm and converted by ACLY into acetyl-CoA, fueling lipid synthesis through ACC, FASN, and SCD-a process regulated by SREBP and HIF-1α. The PI3K/AKT/mTOR signaling axis further drives metabolic reprogramming by enhancing glucose uptake, glycolysis, and lipogenesis. HIF-1α, stabilized under hypoxia, promotes angiogenesis (via VEGF). To adapt to nutrient scarcity, cancer cells upregulate transporters (ASCT2, MCT4) and alternative pathways such as glutaminolysis and lipid storage. *Abbreviations*: *ACLY* ATP Citrate Lyase, *ASCT2* Alanine-Serine-Cysteine Transporter 2, *GLUT1* Glucose Transporter 1, *G6P* Glucose-6-Phosphate, *HK2* Hexokinase 2, *PEP* Phosphoenolpyruvate, *PKM2* Pyruvate Kinase M2, *LDHA* Lactate Dehydrogenase A, *MCT4* Monocarboxylate Transporter 4, *ACC* Acetyl-CoA Carboxylase, *FASN* Fatty Acid Synthase, *SCD* Stearoyl-CoA Desaturase, *IRS* Insulin Receptor Substrate, *PIP2* Phosphatidylinositol 4,5-Bisphosphate, *PIP3* Phosphatidylinositol 3,4,5-Trisphosphate, *GLS1* Glutaminase 1, *α-KG* Alpha-Ketoglutarate, *Srebp* Sterol Regulatory Element-Binding Protein, *VEGF* Vascular Endothelial Growth Factor

Cancer cells exhibit profound alterations in their metabolic pathways to support rapid proliferation, survival, and metastasis-a phenomenon known as metabolic reprogramming [50]. Unlike normal cells, which primarily rely on oxidative phosphorylation (OXPHOS) for energy production, many cancers shift toward aerobic glycolysis (the Warburg effect), glutaminolysis, and lipid metabolism to meet their heightened biosynthetic and bioenergetic demands [51]. Metabolic reprogramming in senescent cells serves as a critical driver of malignancy through interconnected molecular mechanisms that collectively create a tumor-permissive microenvironment (Fig. 3**)**. Mitochondrial dysfunction and ROS accumulation in senescent cells promoting genomic instability form the foundation of this process. As cells enter senescence, their mitochondria undergo structural and functional deterioration characterized by impaired electron transport chain efficiency, decreased OXPHOS capacity, and leakage of electrons that generate excessive reactive oxygen species (ROS) [52, 53]. This ROS surge, primarily superoxide anions and hydrogen peroxide, overwhelms cellular anti-oxidant defenses, including glutathione depletion, and induces oxidative damage to nuclear and mitochondrial DNA. The resulting double-strand breaks activate DDR pathways, including ATM/ATR and p53, which while initially inducing cell cycle arrest, eventually lead to mutational accumulation due to imperfect repair. Critically, ROS also activates pro-inflammatory transcription factors NF-κB and HIF1α, creating a feedforward loop that amplifies both the SASP and genomic instability [54, 55]. This oxidative environment further modifies lipids and proteins, generating advanced glycation end products (AGEs) that crosslink extracellular matrix components and promote tissue stiffness, a known facilitator of tumor invasion [56, 57]. The shift to glycolysis (Warburg effect) in senescent immune cells impairing anti-tumor responses represents a second key mechanism [58]. Senescent cells, despite adequate oxygen availability, upregulate glucose transporters (GLUT1) and glycolytic enzymes (HK2, LDHA, PKM2) while downregulating pyruvate dehydrogenase (PDH) to limit acetyl-CoA entry into PI3K/AKT/mTOR signaling and stabilization of HIF1α even under normoxic conditions [59]. The consequent lactate overproduction serves dual tumor-promoting roles: extracellular acidification (pH 6.0-6.5) inhibits the cytotoxic activity of tumor-infiltrating lymphocytes and NK cells by impairing perforin/granzyme function, while lactate itself acts as a signaling molecule that induces VEGF secretion to stimulate angiogenesis [60, 61]. Nutrient competition in the TME completes the triad of metabolic malignancy drivers. Senescent cells develop an insatiable appetite for glucose and glutamine, overexpressing glutaminase (GLS1) to convert glutamine into α-ketoglutarate for anaplerotic TCA cycle replenishment [62]. This nutrient scavenging depletes resources for neighboring immune cells while supplying building blocks for tumor growth. The metabolic interplay extends to lipid metabolism, where senescent cells upregulate fatty acid synthase (FASN) and sterol regulatory element-binding proteins (SREBPs) to generate phospholipids for membrane production and lipid droplets for energy storage [63, 64]. These lipid-rich senescent cells then engage in bi-directional crosstalk with tumors through exosomal transfer of miR215p that suppress PTEN in cancer cells, further activating PI3K/AKT signaling [65]. The resulting metabolic symbiosis is reinforced by senescent cell-derived ammonia production from glutaminolysis, which inhibits T-cell proliferation by disrupting arginine metabolism [66, 67]. Collectively, these metabolic adaptations create a vicious cycle where senescent cells reshape the TME into a nutrient-depleted, immunosuppressive niche that actively supports tumor initiation, progression, and therapy resistance while crippling anti-tumor immunity. The convergence of mitochondrial dysfunction, aerobic glycolysis, and nutrient competition establishes metabolic reprogramming as a central pillar of senescence-driven malignancy, offering multiple actionable targets for therapeutic intervention.

#### Immunosenescence

**Fig. 4:**
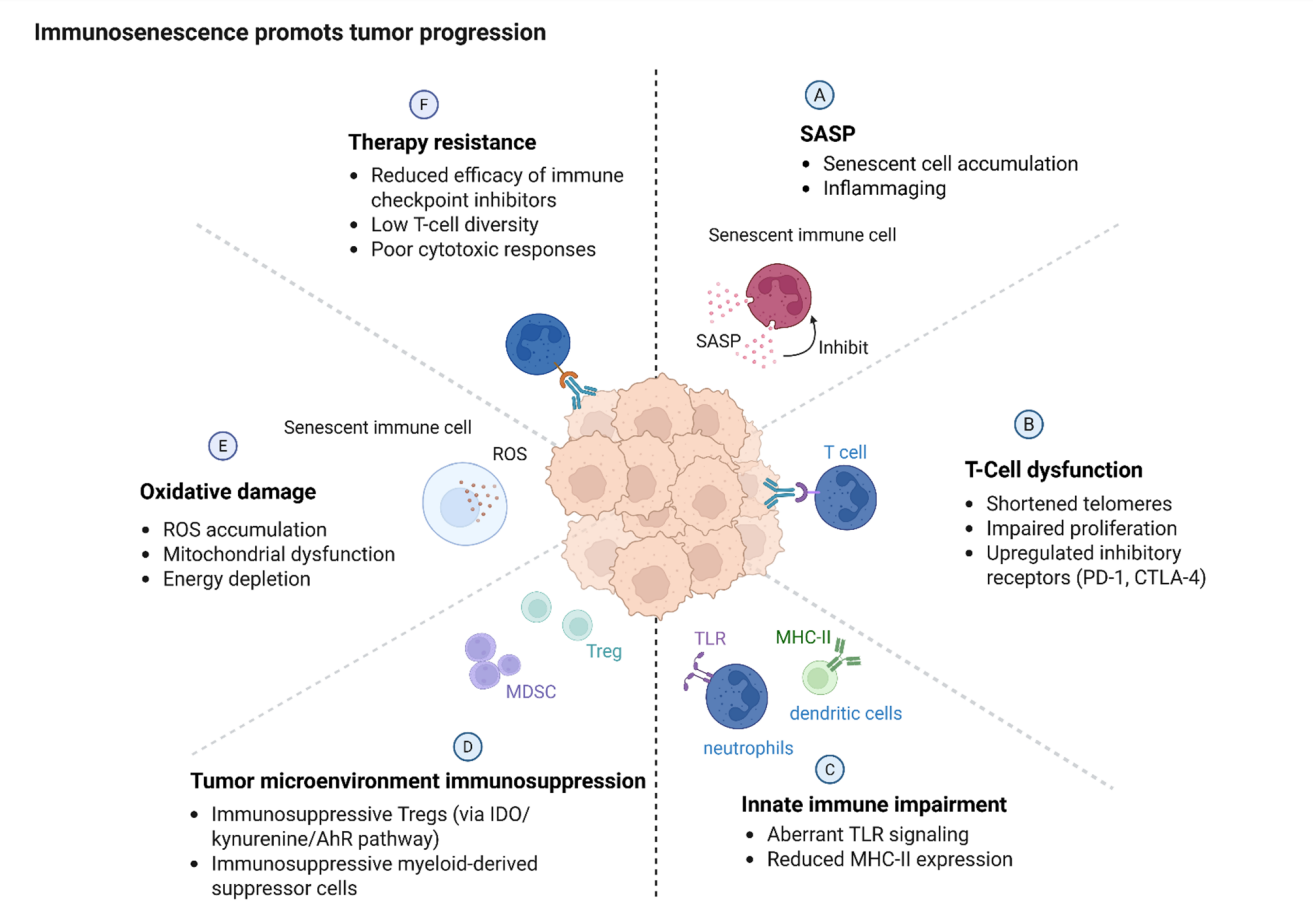
Immunosenescence and its role in promoting tumor progression and therapy resistance. Senescent immune cells accumulate in the tumor microenvironment, exhibiting features such as oxidative damage and T-cell dysfunction. The SASP from these cells drives chronic inflammation and further suppresses immune function. Immunosuppressive cell populations, including Tregs and MDSCs, expand through pathways such as IDO/kynurenine/AhR, creating an immunosuppressive milieu. Innate immunity is also impaired, with aberrant TLR signaling and reduced MHC-II expression on dendritic cells and neutrophils, limiting antigen presentation. These alterations collectively diminish cytotoxic T-cell responses, reduce immune checkpoint inhibitor efficacy, and lower T-cell diversity, fostering therapy resistance. SASP, senescence-associated secretory phenotype. *Abbreviations*: *ROS* Reactive Oxygen Species, *Tregs* Regulatory T cells, *MDSCs* Myeloid-Derived Suppressor Cells, *IDO* Indoleamine 2,3-Dioxygenase, *AhR* Aryl Hydrocarbon Receptor, *TLR* Toll-Like Receptor

Immunosenescence, the age-related decline in immune function, plays a critical role in aging and disease, particularly in cancer progression and therapeutic resistance (Fig. 4**)**. As the immune system ages, it accumulates senescent cells, dysfunctional cells that cease dividing but resist apoptosis, and exhibits SASP [68]. SASP releases pro-inflammatory cytokines, chemokines, and growth factors, creating a state of chronic low-grade inflammation known as “inflammaging”. This environment not only impairs immune surveillance but also promotes tumorigenesis by fostering a pro-tumor microenvironment. For example, in non-small cell lung cancer (NSCLC) and gastric cancer (GC), immune-senescence diminishes the efficacy of immune checkpoint inhibitors (ICIs) due to reduced T-cell diversity, impaired cytotoxic responses, and thymic involution, which limits the production of naïve T cells [69]. Additionally, immune-senescence contributes to immunosuppression in the tumor microenvironment (TME), where senescent immune cells, such as macrophages and neutrophils, fail to clear malignant cells and instead secrete SASP factors that support tumor survival [70]. This dysfunction is exacerbated by psychological stress, which accelerates immune aging through stress hormone-mediated pathways, further compromising cancer immunity [71]. In hepatocellular carcinoma (HCC), immunosenescence undermines immune checkpoint inhibitor (ICI) efficacy, emphasizing the need for biomarkers to predict treatment response in elderly patients [72]. In T cells, aging reduces thymic output (due to thymic involution), diminishing naïve T cell diversity and skewing the repertoire toward memory and exhausted subsets with shortened telomeres and impaired proliferative capacity [73]. Metabolic shifts, such as decreased mitochondrial oxidative phosphorylation and increased glycolysis, further compromise T cell function [74]. Innate immune cells such as macrophages and neutrophils exhibit reduced phagocytosis, impaired antigen presentation, and aberrant toll-like receptor signaling, while dendritic cells show diminished MHC-II expression, hindering adaptive immunity [75]. The TME is reshaped by immune-senescence, with senescent immune cells fostering immunosuppression through increased regulatory T cells (Tregs) and myeloid-derived suppressor cells (MDSCs), which inhibit cytotoxic T and NK cell activity [76]. Additionally, mitochondrial dysfunction and ROS accumulation impair immune cell energetics and amplify oxidative damage [77]. Meanwhile, tryptophan catabolism via indoleamine 2,3-dioxygenase in senescent dendritic cells generates kynurenines that activate aryl hydrocarbon receptor pathways in Tregs, expanding this immunosuppressive population [78]. These molecular disruptions collectively cripple immune surveillance, enabling tumor evasion and reducing the efficacy of ICIs. The interplay between SASP, inflammaging, and metabolic-epigenetic dysregulation underscores immune-senescence as a central driver of age-related immune decline and cancer susceptibility.

#### Epigenetic changes drive malignancy

**Fig. 5:**
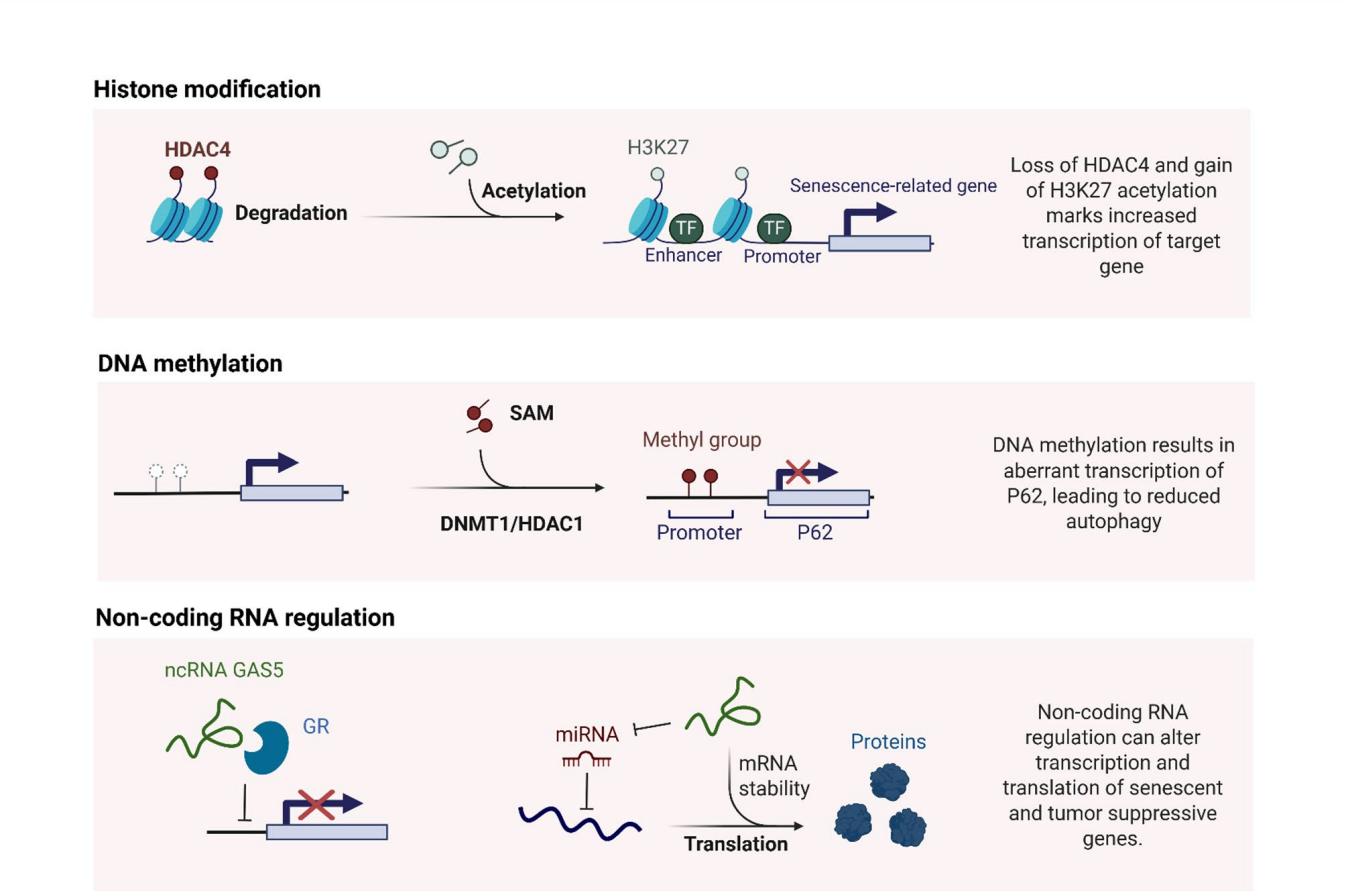
Chromatin remodeling mechanisms in cellular senescence and tumorigenesis. Histone Modification: Degradation of HDAC4 leads to increased H3K27 acetylation at enhancer/promoter regions, activating senescence-related genes. DNA Methylation: DNMT1/HDAC1-mediated promoter methylation of P62 suppresses autophagy, while SAM serves as the methyl donor. Non-coding RNA Regulation: The lncRNA GAS5 modulates transcription, mRNA stability, and translation of senescence/tumor suppressor-related genes through binding regulation and RNA interactions. *Abbreviations*: *SAM * S-Adenosyl Methionine, *DNMT1* DNA Methyltransferase 1, *HDAC4* Istone Deacetylase 4, *GR* Glucocorticoid receptor

Epigenetic changes induced celluar senescence drive malignancy through a complex interplay of histone modification, DNA methylation, and non-coding RNA regulation (Fig. 5**)**. Cellular senescence, initially a tumor-suppressive mechanism, can paradoxically promote malignancy when senescent cells escape growth arrest and acquire pro-tumorigenic phenotypes via epigenetic modifications [79, 80]. Epigenetic modifications silence genes which are critical for immune activation such as CD28 and TCR signaling components while upregulating inhibitory receptors including PD1 and CTLA4 [81]. For instance, senescence-associated chromatin remodeling involves the dynamic modulation of enhancers and super-enhancers by class II-a histone deacetylases, particularly HDAC4, which is degraded during senescence, leading to increased H3K27 acetylation and activation of senescence-related transcriptional programs [82]. In transformed cells, the loss of HDAC4 can reactivate these programs, suggesting its role as an epigenetic checkpoint that, when disrupted, facilitates malignancy [82]. Similarly, poly-comb repressive complex 1 component CBX2 silences tumor suppressor RBL2, inhibiting the DREAM complex and promoting oncogenic signaling such as mTORC1 and E2F in TNBC, illustrating how epigenetic repression of senescence pathways drives proliferation [83]. Epigenetic dysregulation also underpins the heterogeneity of therapy-resistant cancer subclones, as seen in head and neck cancer, where DNMT1 and HDAC1 cooperate to silence p62, conferring radioresistance and enabling clonal survival [84]. Furthermore, the oncogene UHRF1 induces global methylome disordering and heterochromatin loss, triggering senescence but also fostering stemness and HCC development when senescent cells re-enter the cell cycle [85]. SASP components, regulated by epigenetic mechanisms, further create a pro-inflammatory microenvironment that fuels tumor progression [86]. Epigenetic clocks reveal accelerated aging in cancer tissues, yet decelerated aging in noncancerous tissues of BC patients, highlighting systemic epigenetic discordance that may contribute to malignancy [87, 88]. Mitochondrial epigenetic changes, such as altered DNA methylation and miRNA expression, disrupt oxidative phosphorylation and increase ROS production, linking senescence to metabolic reprogramming and tumorigenesis [89]. In ovarian and TNBC, 5AZAdC alter glycosylation patterns and upregulate senescence markers such as p21, but also paradoxically enhance migration, underscoring the dual role of senescence in cancer [90]. Resveratrol modulates histone acetylation to reactivate tumor suppressors such as E-cadherin and p21, thereby inducing senescence and sensitizing TNBC cells to apoptosis [91], while fucoidan inhibits HDAC1, triggering senescence and autophagy in cervical cancer (CC) [92]. The long non-coding RNA GAS5 orchestrates a regulatory mechanism to control senescence-related genes. At the transcriptional level, GAS5 binds to the DNA-binding domain of the glucocorticoid receptor (GR), thereby inhibiting GR-activated genes and facilitating cell cycle arrest through upregulating p21 [93, 94]. Additionally, GAS5 functions as a competitive endogenous RNA by containing multiple miRNA binding sites that sponge oncogenic miRNAs including miR-21, miR-222, and miR-17-5p. This sequestration activity relieves miRNA-mediated repression of tumor suppressor mRNAs, enabling their translation [95]. Telomere maintenance via telomerase reverse transcriptase (TERT) reactivation, often epigenetically regulated, bypasses senescence in pluripotent stem cells and cancers, promoting immortality [96]. Epigenetic reprogramming during aging, including DNA methylation shifts and histone modifications, creates a permissive environment for malignancy by destabilizing chromatin and activating oncogenic pathways [97, 98]. Omega-3 fatty acids such as ALA target epigenetic modifiers including EZH2 and SIRT1 to modulate senescence-related pathways, offering therapeutic potential [99]. In gastrointestinal cancers, pro-senescence therapies leveraging epigenetic modulators aim to counteract progression but must navigate the fine line between tumor suppression and promotion [100]. Overall, senescence-induced epigenetic remodeling drives malignancy through a multifaceted cascade of chromatin reconfiguration, gene silencing/activation, and microenvironmental crosstalk, with implications for targeted therapies.

## Senescence in specific cancers

Cellular senescence plays a dual role in cancer biology, acting as both a tumor-suppressive mechanism and a driver of malignancy. In the early stages, senescence serves as a critical barrier to cancer development by permanently arresting damaged or precancerous cells. However, in established tumors, senescent cells often persist and secrete pro-inflammatory factors that promote tumor growth, angiogenesis, and immune evasion. The impact of senescence varies across cancer types. It may enhance therapy responses in some malignancies, while in others it contributes to treatment resistance and recurrence. This complex duality makes senescence a promising but challenging therapeutic target, requiring cancer-type-specific approaches to either induce or eliminate senescent cells for optimal anti-tumor effects (Table 1**)**.

**Table 1:** Senescence in specific cancers

Organ System	Cancer type	Senescence features	Senescence mechanisms	Therapeutic strategies
Digestive system	Colorectal cancer	- mTOR-mediated senescence (↓SA-β-gal+, IL-6/TNF-α) - p53 stabilization	- USP11 depletion → p53↑ - FGGY knockdown → p53-dependent SAHF	- Oleanolic acid + immune modulators - Berberine derivative B68 + PD-L1 degradation
	Gastric cancer	- Autophagy-senescence crosstalk - IL-8-induced senescent CAFs	- CBX4 SUMOylates YAP1 → Hippo inhibition - lncRNA FGD5-AS1 stabilizes YBX1 → ROS↓	- Exisulind + palbociclib - Metformin to counteract therapy-induced senescence
	Liver cancer	- RAD51 deletion → polyploidization - Metabolic reprogramming (NRF2-FBP1)	- LDHA-mediated H2B lactylation → senescence resistance	- Salidroside + 5FU → mitophagy-associated senescence - Sirolimus (↓SASP)
	Pancreatic cancer	- Senescent CAFs → immunosuppression	- p16/RB and p53/p21 axes - BET inhibitor JQ1 suppresses SASP	- ABT263 - STING/TLR4 agonists + RAS-targeted therapy
Reproductive System	Breast cancer	- Therapy-induced senescence → resistance - SASP remodels TME	- NOTCH1 inhibition → immunogenicity↑ - p53/p21 and CDK4/6-RB pathways	- CDK4/6 inhibitors + lysosomotropic agents - ABT263
	Prostate cancer	- Androgen therapy (SAL) → H2AJ↑ → senescence	- ARv7 activates SKP2 → p27 degradation → senescence escape	- SKP2 inhibitors - PARP inhibitors + senescence inducers
	Ovarian cancer	- PARPi-induced SASP → immune recruitment - PGCCs with stemness post-senescence	- PART1 lncRNA impairs mitophagy → senescence	- Dasatinib/quercetin - PARPi + immunotherapy
Urinary system	Bladder cancer	- Mitophagy (PINK1/DARS2) → senescence suppression	- AURKB/MAD2L2 → p53 DDR inhibition	- CDK4/6 inhibitors (palbociclib) - Senolytics for SASP
	Kidney cancer	- TNFSF15↓ → immune dysfunction - Resveratrol → CCNB1 destabilization	- PML inhibition → p53 restoration	- Osalmid + navitoclax - Arsenic trioxide (PML inhibitor)
Respiratory system	Lung cancer	- T cell senescence (SLC2A1/TNS4 signature) - Fibroblast senescence → invasion	- CNOT3 knockdown → p21↑ - Hypoxia (HIFs) → chronic senescence	- SARMs → senescence induction - Engineered BMSCs (sPD-1 + IFN-γ)
Nervous system	Brain cancer	- SASP (IL-6/IL-1β) → tumor progression - Radiation-induced astrocyte senescence	- TRAF7 knockdown → KLF4-mediated senescence	- Vagus nerve stimulation (↓SASP) - Gambogic acid (BMII degradation)
Hematologic System	Blood cancer	- Premature aging in survivors (↑IL-6/CRP) - β-arrestin1 → RAS-p16 senescence	- MYC/API-driven senescence escape	- BCL2/JAK-STAT inhibitors - Senolytics for late effects
Endocrine System	Thyroid cancer	- AR activation → p16/p21↑ - D2-driven dedifferentiation	- ZNF24 inhibits Wnt/β-catenin → senescence	- CDK2 inhibitors (lenvatinib resistance) - ZNF24 targeting

*Abbreviation*: *SA-β-gal* Senescence-Associated β-galactosidase, *SASP* Senescence-Associated Secretory Phenotype, *TME* Tumor Microenvironment, *CAFs* Cancer-Associated Fibroblasts, *DDR* DNA Damage Response, *PGCCs* Polyploid Giant Cancer Cells, *SAHF* Senescence-Associated Heterochromatin Foci

### Digestive system

#### Colorectal cancer

Colorectal cancer (CRC) is one of the most common tumors worldwide, causing a prominent global health burden. A series of studies investigate the complex role of cellular senescence in CRC pathogenesis, therapeutic response, and TME modulation. Oleanolic acid mitigates 5-FU-induced intestinal damage by suppressing mTOR-mediated senescence (reducing SA-β-gal + cells and SASPs such as IL6/TNF-α) [101], while berberine derivative B68 simultaneously induces senescence via BMI1 inhibition and enhances immune clearance through CSN5-mediated PD-L1 degradation [102]. The MDM2-p53-p21 axis emerges as critical, with USP11 depletion promoting senescence through p53 stabilization [103] and FGGY knockdown activating p53-dependent senescence-associated heterochromatin foci formation (evidenced by increased H3K9me3 and HP1γ) [104]. Epigenetic regulation via RNA pseudouridine (Ψ) modification correlates with senescence through mTOR/TGF-β pathways, with the Psi Score serving as a predictive biomarker for immunotherapy response [105].

TIS studies reveal chemotherapeutic agents (SN38, etoposide, doxorubicin) induce senescence accompanied by HBP/O-GlcNAcylation downregulation, while OGT inhibition shifts senescence to apoptosis, suggesting combinatorial strategies [106]. Single-cell analyses identify UISGF3-related genes (including HSH2D) and JAK/STAT signaling as drivers of doxorubicin resistance in proliferative persister cells [107]. Prognostically, senescence-related gene signatures stratify CRC patients (HR = 2.73, *p* = 6.4E-16), with high-score tumors exhibiting immunosuppressive CXCL2/3-CCL3/4-mediated microenvironments [108]. Ferroptosis-immunosenescence crosstalk highlights ROS-mediated lipid peroxidation as a nexus between iron dysregulation and immune dysfunction [109]. CVBD induces autophagy/senescence via CCT3/YAP inhibition, while curcumin suppresses EGR1-mediated transcriptional repression of TERT/SIRT6, promoting senescence [110].

Collectively, these findings underscore senescence as a double-edged sword in CRC while inducible senescence offers therapeutic potential, its association with immune evasion and TIS escape necessitates precision approaches combining senescence inducers with immune modulators or O-GlcNAcylation inhibitors, guided by epigenetic (Psi Score) and transcriptomic (senescence signatures) biomarkers to optimize therapeutic efficacy.

#### Gastric cancer

GC remains a formidable global health challenge and emerging evidence highlights the critical role of cellular senescence in tumor progression, therapeutic resistance, and immune modulation. Recent studies demonstrate that GC exhibits distinct senescence-related molecular subtypes, which correlate with prognosis and treatment response. For instance, a 14-gene prognostic signature integrating autophagy and senescence-related genes effectively stratifies GC patients into risk groups, with high-risk patients showing elevated immune checkpoint expression, stromal scores, and immune evasion tendencies, while low-risk patients display microsatellite instability-high status and higher tumor mutational burden, suggesting enhanced immunotherapy responsiveness [111]. Similarly, the GC-specific Senescence Score identifies patients with upregulated senescence markers and immunosuppressive features such as PVR-CD96 signaling and IL6/CXCL12-secreting CAFs, while drug repositioning reveals exisulind as a potent senolytic agent that synergizes with palbociclib to eliminate senescent GC cells [112].

Mechanistically, senescence in GC is regulated by diverse pathways. The SUMO E3 ligase CBX4 promotes chemoresistance by stabilizing YAP1 via SUMOylation, thereby inhibiting the Hippo pathway and senescence induction [113]. Conversely, the lncRNA FGD5AS1, transcriptionally activated by ZEB1, suppresses senescence and ROS production by binding and stabilizing YBX1, while its knockdown enhances cisplatin sensitivity [114]. Senescence-associated lncRNAs such as LINC01579 and AP000695.2 further refine prognostic models, with high-risk signatures correlating with poor survival, M2 macrophage infiltration, and T-cell dysfunction [115, 116]. Notably, diffuse-type GC exhibits unique senescence dynamics: DGC cells induce senescent CAFs via IL8 secretion, fostering peritoneal metastasis [117], while aurora kinase inhibitors trigger senescence in DGC organoids, leading to MCP1/CCL2mediated M2 macrophage polarization and immune suppression [118].

Therapeutic strategies targeting senescence show promise but require precision. Natural compounds such as metformin counteract chemotherapy-induced senescence while enhancing apoptosis, whereas dasatinib-edaravone combinations modulate the SASP and immune microenvironment [119]. Nanomedicine approaches aim to overcome the delivery limitations of these agents. Additionally, risk models integrating lysosomal and senescence markers such as MMP12 or drug sensitivity profiles including ML210 and temsirolimus guide personalized therapy [120].

In conclusion, cellular senescence in GC is a double-edged sword, with pro-tumorigenic effects such as immunosuppression and chemoresistance countered by therapeutic opportunities including senolysis and immune activation. Senescence-targeted combinational therapies may transform GC management by harnessing senescence as a therapeutic vulnerability.

#### Liver cancer

Cellular senescence in HCC exhibits a paradoxical nature, functioning as both a tumor-suppressive mechanism and a contributor to malignant progression.

The RAD51 recombinase maintains genomic stability in hepatocytes, with its deletion inducing premature senescence, polyploidization, and fibrosis while paradoxically conferring resistance to chemical hepatocarcinogenesis [121]. Metabolic reprogramming through the NRF2-FBP1-AKT-p53 axis enables senescence escape in MASH-associated HCC, with FBP1 silencing promoting progenitor cell proliferation [122]. Epigenetic modifications via LDHA-mediated histone H2B lactylation (K58) on NDRG1 establish senescence-resistant sub-populations with enhanced metastatic potential [123]. The combination of salidroside with 5-FU demonstrates synergistic effects by inducing mitophagy-associated senescence through YIPF5 inhibition, offering a potential solution to 5-FU resistance in metabolic dysfunction-associated HCC [124]. Simultaneously, the inhibition of the EZH2/MCM complex/hTERT axis and DUSP3 knockdown (which activates Notch1-mediated senescence) provide alternative routes to trigger irreversible growth arrest in HCC cells [125]. Sirolimus has shown chemoprophylactic potential by reducing SASP factors (TNF-α, IL1β) and HCC nodule formation in preclinical models, while CBX4 inhibition restores Hippo pathway activity to overcome chemoresistance [126]. Immunosenescence modulation through Yangyin Fuzheng Jiedu Prescription (YFJP) ameliorates CD8 + T cell dysfunction via FoxO1 upregulation, potentially enhancing checkpoint inhibitor efficacy [127]. Machine learning analysis of single-cell RNA sequencing data has identified eight HCC cell senescence markers (NOX4, BIRC5, E2F1, CD34, KIF2C, AURKA, CDK1, and GMNN) that classify patients into molecular subtypes with distinct mutation profiles and treatment responses [128]. A robust 14-gene senescence signature further stratifies patients by immune evasion potential and microsatellite instability status, while CCNB1 has emerged as a particularly promising diagnostic biomarker (AUC = 0.89) that correlates with M0 macrophage infiltration and cell cycle dysregulation [129]. Together, HCC senescence paradoxically suppresses and promotes cancer, with metabolic, epigenetic, and immune interplay offering novel therapeutic targets and biomarkers.

#### Pancreatic cancer

Pancreatic ductal adenocarcinoma (PDAC) is a highly aggressive malignancy with a notoriously poor prognosis, largely due to its complex and desmoplastic TME. A key feature of PDAC is the abundance of CAFs, which exhibit significant heterogeneity and play diverse roles in tumor progression. Recent studies have identified a distinct subpopulation of senescent myofibroblastic CAFs (SenCAFs) that localize near tumor ducts and accumulate during PDAC progression [130]. These SenCAFs contribute to immune suppression and chemoresistance, as demonstrated in preclinical models where their depletion alleviated macrophage-mediated immunosuppression, delayed tumor growth, and enhanced chemotherapy efficacy [131].

Further investigations reveal that cellular senescence in PDAC has dual roles: while it can act as a tumor-suppressive mechanism by halting proliferation, it also promotes a pro-tumorigenic environment through the SASP. For instance, the BET inhibitor JQ1 induces senescence in PDAC cells but suppresses SASP, highlighting the nuanced interplay between senescence and tumor progression [132]. Additionally, targeting senescence-related pathways, such as the p16/RB and p53/p21 axes, or employing senolytic agents such as ABT263, has shown promise in overcoming therapy resistance [133–135].

Emerging strategies, such as nanoparticle-mediated delivery of STING and TLR4 agonists combined with RAS-targeted therapies, aim to remodel the immunosuppressive TME and activate durable anti-tumor immune responses [136]. Epigenetic regulators such as EZH2 and noncoding RNAs such as circHIF1α further modulate senescence and immune evasion, offering new therapeutic targets [137, 138]. Collectively, these findings underscore the critical role of senescence in PDAC heterogeneity and resistance, paving the way for innovative combination therapies to improve patient outcomes.

#### Esophageal cancer

Esophageal cancer (ESCA) is profoundly influenced by cellular senescence, a process that serves as both a tumor-suppressive mechanism and a contributor to therapy resistance and tumor progression. In esophageal squamous cell carcinoma, OIS is modulated by factors such as RECQL4, whose depletion induces senescence via DDR impairment, oxidative stress, and G0/G1 arrest, highlighting its role in ESCA progression [139]. Similarly, the RNA-binding protein FXR1 promotes ESCA aggressiveness by destabilizing tumor-suppressive mRNAs (PDZK1IP1, ATOH8), and its knockdown triggers senescence, suggesting therapeutic potential [140]. Oxidative and endoplasmic reticulum stress further drive senescence in ESCA, with genes such as TFRC activating HIF1α and NOTCH pathways to promote proliferation while senescence-related pathways such as p53 signaling are enriched in ESCA-specific gene networks [141, 142]. Therapeutic interventions often exploit senescence: the survivin inhibitor YM155 enhances radiosensitivity by switching radiation-induced senescence to apoptosis in p21-dependent ESCC cells [143], while CDK4/6 inhibitors such as palbociclib induce senescence and are being explored for ESCA treatment [144]. Conversely, RRM2 inhibition by Osalmid synergizes with radiotherapy to amplify DNA damage and senescence, overcoming radioresistance [145]. Senescence also shapes the TME. Radiation-induced stromal fibroblasts exhibit SASP, which promotes EC cell proliferation and invasion via NF-κB; this is mitigated by metformin, underscoring SASP’s protumor role [146]. Immune evasion in ESCA is linked to senescence-related risk scores, where high CSRS correlates with poor prognosis and immunotherapy resistance, driven by genes such as IGFBP1 and SOX5 [147]. Additionally, CD59 overexpression confers radio-resistance by suppressing senescence via Src kinase activation, making it a predictive biomarker [148]. Natural compounds such as gypenoside L induce senescence in ESCA cells via p38/ERK and NF-κB pathways, enhancing chemosensitivity [149], while sirtuins exhibit context-dependent roles in ESCA senescence and metabolism [150]. Notably, eosinophilic esophagitis inflammation paradoxically limits ESCA tumorigenesis by inducing suprabasal cell senescence, contrasting with GERD’s cancer-promoting effects [151]. Senescence-related biomarkers (ASPM, KIF11) and pathways (p53, cellular senescence) are recurrent in ESCA genomics, offering diagnostic and therapeutic targets [142]. Collectively, senescence in ESCA is a double-edged sword, warranting tailored strategies-such as senolytics to eliminate harmful senescent cells or SASP modulators to disrupt pro-tumor signaling to improve outcomes in this aggressive malignancy [152–155].

### Reproductive system

#### Breast cancer

Breast cancer (BC) is a complex disease where cellular senescence plays a dual role, influencing both tumor progression and treatment outcomes. Senescence, a state of irreversible cell cycle arrest, is implicated in BC through various mechanisms, including TIS, which can paradoxically contribute to drug resistance and relapse [156]. For instance, ADGRF1 switches from tumor-promoting to tumor-suppressive functions upon interaction with laminin-111, enhancing sensitivity to anti-HER2 drugs in HER2 + BC [157]. Similarly, BRCA1 regulates kidney injury responses but also influences BC cell senescence and fibrosis, highlighting its context-dependent role [158]. Senescence is also linked to immune evasion, as senescent T cells, particularly CD28-CD57 + phenotypes, are elevated in BC patients and correlate with age-related immune dysfunction [159]. The p53 pathway is central to senescence regulation, with *Ziziphus nummularia* extract inhibiting BC cell proliferation by modulating senescence-related genes such as Cyc-E and p21 [160]. Kinds of senescence-related proteins and mechanisms have been delineated in BC. NOTCH1 inhibition induces senescence and enhances immunogenicity in TNBC, improving responses to immune checkpoint inhibitors [161]. HGH1 promotes BC growth via the PI3K/AKT/NF-κB pathway, linking senescence to poor prognosis [162], while Lithocholic Acid induces ferroptosis in TNBC through iron metabolism and autophagy [163]. TFDP1, a target of topotecan, inhibits senescence in TNBC, suggesting that combined senolytic therapies could improve outcomes [164]. Telomere maintenance and senescence-related gene signatures offer prognostic value, revealing tumor heterogeneity and immune interactions [165]. Metallodrugs such as PtL_2_ induce senescence and cell cycle arrest, providing alternatives to conventional therapies [166]. CDK4/6 inhibitors induce senescence but face resistance, which can be overcome by lysosomotropic agents [167]. Proton beam irradiation triggers senescence and DNA damage in BC cells, affecting metastatic potential [168]. Combining doxorubicin with venetoclax reduces senescence and enhances apoptosis in TNBC, independent of p53 status [169]. C-Myc, overexpressed in TNBC, drives senescence-related pathways, making it a therapeutic target [170]. Genistein synergizes with chemotherapy to induce senescence, particularly in ER + BC [171]. Salkosaponin A induces senescence via ROS-mediated PI3K/Akt inhibition [172]. SASP shapes the tumor microenvironment, influencing BC progression and treatment responses [173]. Finally, RUNX1-PDGF-BB axis activation drives CDK4/6 resistance, highlighting the need for targeted strategies [174]. Together, these studies underscore senescence’s multifaceted role in BC, offering insights into novel therapeutic approaches.

#### Prostate cancer

Prostate cancer (PCa) is a significant public health issue, particularly in developed countries, where the androgen receptor (AR) plays a central role in both normal prostate development and cancer progression. Recent research has explored the therapeutic potential of supraphysiological androgen levels (SAL) in bipolar androgen therapy, which induces cellular senescence in AR + PCa cells, thereby suppressing tumor growth. Key findings reveal that SAL upregulates histone variant H2AJ, a direct AR target gene that promotes cellular senescence and inhibits mesenchymal transition, as evidenced by reduced H2AJ expression in metastatic tumors [175]. Additionally, SAL modulates the expression of long non-coding RNAs such as MIR503HG, which is suppressed by SAL and associated with metastatic progression and poor survival. MIR503HG represses senescence by regulating the AKT-p70S6K and p15(INK4b)-pRb pathways, while its downregulation enhances BRCA2 depletion, suggesting potential synergy with PARP inhibitors [176]. Another lncRNA, ADAMTS9AS2, acts as an AR coactivator, whereas PART1 serves as a corepressor, both influencing AR signaling and senescence [177]. Meanwhile, the clock gene BHLHE40 and its target LYL1 form a regulatory loop with p27kip1 to mediate SAL-induced senescence, with LYL1 knockdown exacerbating senescence through p27kip1 upregulation [178]. CircRPS6KC1, an m6A-modified circular RNA, also impacts senescence by sponging miR761 to regulate FOXM1/PCNA/p21 signaling [179]. Senescence-related mitochondrial dysfunction leads to extracellular mtDNA release via VDAC channels, which enhances immunosuppressive PMN-MDSC activity through cGAS-STING-NF-κB signaling, promoting tumor progression [180]. Radiotherapy-induced senescence is further modulated by ALDH1A3, which regulates SASP via cGAS-STING, while cytosolic ATR activation in senescent cells impairs antigen presentation and immune checkpoint blockade efficacy [41]. In castration-resistant PCa, AR variant ARv7 drives escape from ADT-induced senescence by activating SKP2, which degrades p27 to restart cell proliferation, highlighting SKP2 inhibition as a therapeutic strategy [181]. Collectively, the studies underscore the complexity of androgen signaling, senescence regulation, and immune evasion in PCa, offering novel targets such as H2AJ, MIR503HG, ARv7/SKP2, and VDAC for combination therapies to improve treatment outcomes.

#### Cervical cancer

Cervical cancer (CC) is intricately linked to cellular senescence, a process influenced by human papillomavirus infection, oncogenic signaling, and therapeutic interventions. HPV, particularly high-risk types, induces senescence in cervical epithelial cells, marked by p16 overexpression, enlarged nuclei, and inflammatory infiltration, which may serve as early diagnostic indicators [182]. OIS is observed in premalignant lesions, with downregulation of Lamin B1 and altered expression of senescence markers such as TP53, IL1A, and CCL2, suggesting its role in early carcinogenesis [183, 184]. However, senescence has a dual role: while it initially suppresses tumorigenesis, senescent cells in the TME promote immune evasion and treatment resistance. For instance, CCRT-resistant CC cells drive CD8 + T cell senescence via ACKR2 and TGF-β, facilitating recurrence [185]. Similarly, exosomal mortalin, stabilized by METTL3-mediated m6A methylation, suppresses p53-mediated senescence, enhancing CC progression [186]. SASP components, such as elevated CXCL8 and TGF-β1, remodel the TME, fostering inflammation and metastasis [70, 184]. Therapeutic strategies targeting senescence are emerging. DOC2B, delivered via extracellular vesicles (EVs), induces senescence in CC cells by elevating ROS and inhibiting AKT/ERK pathways, suppressing aggressiveness [187]. miR34b promotes senescence by targeting TWIST1, while the C14MC miRNA cluster reactivation induces senescence via metabolic reprogramming and PDK3 inhibition [188, 189]. Senolytics such as ABT263 and ABT199 eliminate therapy-induced senescent cells, restoring cisplatin sensitivity [190]. Nanopore sequencing identifies active HPV integrants, enabling CRISPR/Cas9 targeting to induce senescence and curb proliferation [191]. Placental EVs, carrying senescence-associated miRNAs, exhibit anti-tumor effects by inducing necrotic and senescent phenotypes in CC cells [192]. Conversely, RRM2 overexpression in gynecological cancers, including CC, suppresses senescence, promoting proliferation and chemoresistance [193]. Radiation therapy exacerbates senescence in locally advanced CC, with IL1R1 inhibition (anakinra) proposed to mitigate recurrence [194]. SOX6 induces senescence via TGF-β2-Smad2/3-p21 pathways, though senescent cells resist cisplatin, necessitating senolytic combinations [190]. SNAI2 overexpression in HPV-negative CC reduces senescence, correlating with dormancy and poor prognosis [195]. These findings underscore senescence as a double-edged sword in CC, highlighting its potential as a biomarker and therapeutic target. Strategies combining senolytics, immunotherapy, and precision targeting of HPV integrants or SASP components may improve outcomes, particularly in resistant or advanced diseases [70, 196, 197].

#### Ovarian cancer

Ovarian cancer (OC), particularly high-grade serous OC, is a lethal malignancy often diagnosed at advanced stages with poor prognosis due to chemoresistance and recurrence [198, 199]. Cellular senescence, a state of irreversible cell cycle arrest, plays a dual role in OC, acting as both a tumor suppressor and a contributor to therapy resistance and TME remodeling [200, 201]. Senescence can be induced by oncogenic stress such as RAD51D and TP53 mutations, DDR activation, or chemotherapy agents such as cisplatin and PARP inhibitors, leading to upregulation of senescence-associated biomarkers such as β-galactosidase (SA-β-gal) and p16/p21 [202–204]. For instance, PARP inhibitors such as olaparib induce senescence in homologous recombination-proficient OC by triggering SASP and recruiting immune cells, suggesting a novel immunomodulatory mechanism [205, 206]. However, senescence also promotes chemoresistance; polyploid giant cancer cells (PGCCs) formed under docetaxel treatment exhibit senescence phenotypes and stem-like properties, contributing to relapse [207]. Similarly, carboplatin/paclitaxel-induced senescence in peritoneal fibroblasts and mesothelial cells paradoxically fuels OC progression via cytokine secretion [208, 209]. Epigenetic regulation further modulates OC senescence, with lncRNAs such as sin-lncRNA and PART1, along with senescence-related gene signatures such as SGKL/VEGFA and IGFBP5, influence metabolic reprogramming, immune evasion, and drug sensitivity [200, 210]. For example, PART1 knockdown impairs mitophagy via PHB2 degradation, driving senescence and PARPi resistance [211], while CDK4/6 inhibitors such as palbociclib induce p53 acetylation-dependent senescence in HGSC [204]. The TME’s role is critical: senescent OC cells and adipose-derived stem cells (ADSCs) create a pro-inflammatory niche via SASP such as IL6 and CXCL10, recruiting immunosuppressive M2 macrophages and exhausted CD8 + T cells, while impairing cytotoxic T-cell function [212]. Senolytic agents such as dasatinib and quercetin counteract these effects by eliminating senescent ADSCs, reducing metastasis, and restoring glucose homeostasis [209]. Immune senescence markers such as CD57 + CD8 + T cells correlate with poor prognosis, whereas PD1 + CD8 + T cells associate with better outcomes, highlighting the interplay between senescence and immune dysfunction [213, 214]. Mitochondrial and metabolic dysregulation, such as FeS cluster deficiency or lipid metabolism alterations, further links senescence to ferroptosis and OC progression [215, 216]. Therapeutic strategies targeting senescence include combining PARPi with immunotherapy, exploiting SASP-mediated immune activation [217], or using probes such as gal-HCA and HDQ-NA-AFU-Gal to detect senescence-associated enzymes [218]. Despite its tumor-suppressive potential, senescence in OC often exacerbates malignancy through metabolic rewiring, stemness acquisition, and TME crosstalk, necessitating precision approaches to harness its benefits while mitigating adverse effects.

#### Endometrial cancer

Endometrial cancer (EC) is intricately linked to cellular senescence, a process that can both suppress tumorigenesis and drive cancer progression depending on context. Telomerase activity, mediated by TERT, is elevated in EC and crucial for maintaining telomere length to avoid senescence, with progesterone inhibiting telomerase, offering a potential therapeutic avenue [219, 220]. Senescence-related pathways are implicated in EC metastasis, as serum protein profiling reveals associations between lymph node metastases and senescence-inducing pathways such as PTEN loss and PI3K/AKT activation [221]. Natural compounds such as methoxyeugenol induce senescence in EC cells by elevating ROS, disrupting mitochondrial function, and upregulating p53/p21 while downregulating CDK4/6, highlighting its anti-cancer potential [222]. Combination therapies targeting EphA2 and histone deacetylase such as panobinostat synergistically enhance DNA damage and apoptosis while downregulating senescence-related survival pathways [223]. Cisplatin resistance in EC can be overcome by combining it with repurposed drugs such as resveratrol or trichostatin A, which induce senescence and autophagy [224]. Genetic studies reveal bi-directional links between EC and BC, with cellular senescence emerging as a shared mechanistic pathway [225]. Tumor suppressors such as IGFBPrP1 and FOXA1 promote senescence in EC by inhibiting ERK signaling and activating p16 INK4a via the AKT pathway, respectively [226, 227]. Similarly, miR365 suppresses EC progression by targeting oncogenes EZH2 and FOS, inducing senescence and enhancing chemosensitivity [228]. Hormonal therapies, such as megestrol acetate, trigger irreversible G1 arrest and senescence via PRB/FOXO1mediated upregulation of p21 and p16 [229]. SWI/SNF chromatin remodelers, particularly ARID1A/ARID1B, regulate senescence, with their loss driving EC carcinogenesis [230]. Machine learning models leveraging senescence-related genes stratify EC patients into high and low-risk groups, underscoring senescence as a prognostic biomarker [231]. RRM2, overexpressed in EC, promotes tumor progression by bypassing senescence via p53 and Akt/mTOR pathways [193]. The MSC marker SUSD2 counteracts senescence in EC, with its silencing inducing SMAD2/3-mediated cell death [232]. Inhibitor of growth protein 2 exhibits altered expression in EC, suggesting a role in senescence and tumor suppression [233]. Additionally, bioactive exosomes from aurea helianthus extract induce senescence by impairing mitochondrial function and enhancing autophagy [234]. Collectively, these studies demonstrate that senescence plays a dual role in EC, serving as a barrier to tumorigenesis when activated by suppressors such as IGFBP-rP1 or FOXA1, yet contributing to therapy resistance and metastasis when dysregulated. Targeting senescence pathways, through telomerase inhibition, natural compounds, or combination therapies, holds promise for improving EC treatment.

### Urinary system

#### Bladder cancer

Bladder cancer (BLCA) is closely linked to cellular senescence, a process that influences tumor progression, therapy resistance, and immune modulation. Senescence in BLCA is regulated by diverse molecular mechanisms, including mitophagy, DDR, and oncogenic signaling. For instance, DARS2 promotes BLCA progression by enhancing PINK1-mediated mitophagy, which suppresses senescence and drives cell proliferation [235]. Conversely, AURKB and MAD2L2 synergistically inhibit senescence by downregulating the p53 DDR pathway, facilitating BLCA cell survival and metastasis [236]. Similarly, METTL13 activates the PI3K/AKT/mTOR pathway to bypass senescence, enhancing BLCA cell viability and invasion [237]. The tumor suppressor C2orf40, often downregulated in BLCA, is associated with poor prognosis and senescence induction, highlighting its potential as a pan-cancer biomarker [238]. Environmental factors such as tobacco smoke and disinfection byproducts also modulate senescence; the smoking carcinogen 4-aminobiphenyl upregulates ELAVL1 to inhibit senescence via autophagy, while HAMs induce senescence through p16 and p53/p21 pathways, implicating these mechanisms in BLCA etiology [239, 240]. Therapeutic interventions often exploit senescence: HSP90 inhibitor BIIB021 alters cancer-related genes, including those involved in senescence, to suppress BLCA growth [241], while CDK4/6 inhibitors such as palbociclib induce G1 arrest and senescence in RB + BLCA cells [242]. Plasma-activated solution synergizes with mitomycin C to trigger senescence via ROS-mediated DNA damage [243], and PRPF19, an E3 ligase, correlates with BLCA senescence and stemness, offering prognostic value [244]. Senescence also shapes the TME. The LIG1 gene, overexpressed in BLCA, promotes proliferation and immune evasion while influencing senescence and p53 signaling [245]. Sex disparities in BLCA are partly driven by senescence-like neutrophils, which accumulate in males due to microbiome differences, fostering immunosuppression [246]. Senescence-associated molecular subtypes, such as those identified by Senescore, reveal that high senescence correlates with basal BLCA, immune infiltration, and SASP factors, predicting poorer outcomes but better immunotherapy response [247]. Similarly, senescence-related gene signatures stratify BLCA patients into risk groups, with low-risk patients showing better chemosensitivity [248]. STING-mediated immune senescence further influences BLCA prognosis and immunotherapy efficacy [249]. Non-coding RNAs such as SNHG15, regulated by super-enhancers, drive BLCA malignancy by bypassing senescence via the Wnt/β-catenin pathway [250], while YARS1 links senescence to ferroptosis and immune infiltration [251]. Despite its tumor-suppressive role, senescence can paradoxically aid BLCA progression through SASP-mediated TME remodeling, as seen in chemotherapy-induced senescence, which contributes to cognitive impairment via microglial and endothelial senescence [252]. These findings underscore senescence as a double-edged sword in BLCA, with therapeutic potential via senolytics [247] or combinatory approaches targeting senescence pathways [237, 243]. Understanding these mechanisms could refine personalized therapies, improve prognostic models, and mitigate treatment-related senescence in BLCA management.

#### Kidney cancer

Clear cell renal cell carcinoma (ccRCC) poses significant therapeutic challenges, particularly in elderly patients, where ICI responses are often diminished. Research highlights the TNFSF15 gene as a key age-related biomarker, with lower expression in older ccRCC patients correlating with poor survival and advanced pathological stages. This gene’s interaction with senescence and immune pathways underscores its potential as a therapeutic target [253]. Meanwhile, resveratrol emerges as a promising agent, inducing ccRCC cell senescence by destabilizing CCNB1 mRNA via RBM15 and inhibiting EP300/CBP, thereby suppressing tumor progression [254]. Single-cell and bulk RNA sequencing further reveal distinct senescence-related molecular subtypes in ccRCC, with DUSP1 identified as a feasible biomarker for prognostic stratification [255]. These subtypes, such as the aggressive SPCS2, exhibit dysregulated metabolic and immune pathways, poor ICI response, and frequent genomic alterations, emphasizing the role of senescence regulators such as MECP2 in tumor progression [256]. Another study uncovers PML as a critical oncogenic dependency in ccRCC, where its inhibition by arsenic trioxide restores p53 activity, triggering senescence and chemosensitivity [257]. Senescence-related immune dysregulation is further explored through the SenMayo classification, which links high immune activity to poor prognosis, suggesting that aging-associated immune dysfunction drives resistance [258]. Combinatorial therapies also show promise: osalmid, targeting RRM2 to induce senescence, synergizes with the senolytic navitoclax to exploit BCLXL dependence, effectively reducing tumor growth in preclinical models [259]. Additionally, celastrol mitigates senescence-driven invasion and stemness in ccRCC by downregulating CAV1, a mediator of SASP, offering a novel strategy to counteract pro-tumorigenic effects [260]. Metabolic reprogramming in ccRCC is intricately tied to senescence, as demonstrated by the senescence-metabolism risk model, where PTGER4 emerges as a regulator of lipid metabolism and proliferation [261]. Finally, hierarchical clustering identifies two senescence-based ccRCC subtypes, with the C2 subtype associated with aggressive features, p53 pathway dysregulation, and reduced immunotherapy efficacy [262]. Collectively, these findings illuminate the dual role of senescence in ccRCC-both as a tumor-suppressive mechanism and a driver of resistance.

### Respiratory system: lung cancer

Non-small cell lung cancer (NSCLC) is a leading cause of cancer-related deaths, with T cell senescence playing a critical role in impairing antitumor immunity. Recent studies identified a prognostic gene signature (SLC2A1, TNS4, GGTLC1) linked to T cell senescence, which predicts immunotherapy response and poor survival in NSCLC patients. Notably, GGTLC1 overexpression suppresses T cell senescence, suggesting its therapeutic potential [263]. Meanwhile, selective androgen receptor modulators such as S4 show promise in NSCLC treatment by inhibiting proliferation, migration, and colony formation in A549 cells while upregulating proapoptotic genes (BAX, PUMA) and inducing senescence [264]. Metabolic dysregulation also influences NSCLC progression; silencing LDHB disrupts nucleotide metabolism, exacerbates DNA damage, and enhances radiotherapy sensitivity by promoting p21-mediated senescence [33]. The tumor microenvironment further contributes to NSCLC aggressiveness, as senescent fibroblasts in idiopathic pulmonary fibrosis secrete exosomal MMP1, activating PAR1/PI3K-AKT-mTOR signaling to drive cancer proliferation and invasion-a pathway targetable by PAR1 inhibitors [265]. Additionally, molecular regulators such as CNOT3 and PAD2 modulate senescence: CNOT3 knockdown elevates p21 and SASP factors, while PAD2 inhibition suppresses Nrf2/HO1/AKT signaling, impairing tumor growth and amplifying IL6/p53-dependent senescence [266]. Hypoxia-inducible factors and senescence exhibit bi-directional crosstalk, where chronic HIF activation exacerbates pulmonary aging and cancer, highlighting the therapeutic potential of HIF modulators and senolytics [267]. Novel lncRNAs such as lncRAINY also emerge as radiosensitizers, inducing chromatin remodeling and senescence via CDC6/CDC25A regulation [268]. Immunotherapy advances include engineered bone marrow-derived mesenchymal stem cells (BMSCs) delivering sPD1 and IFNγ, which enhance T cell activation, suppress PI3K/AKT/PD-L1 pathways, and promote tumor cell senescence [269]. Finally, combining senescence-inducing agents such as etoposide with targeted therapies including erlotinib and dasatinib exploits unique vulnerabilities in senescent tumors, offering a strategic approach to overcome drug resistance [270]. Together, these findings underscore the interplay between senescence, metabolism, and immunity in NSCLC, revealing multi-targeted strategies to improve therapeutic outcomes.

### Nervous system: brain tumor

Glioblastoma (GBM) and other gliomas remain highly resistant to current therapies, including ICIs, due to their immunosuppressive TME, blood-brain barrier, and cellular heterogeneity. Emerging strategies aim to reprogram the TME by targeting SASP components, such as IL6, IL1β, and TNF-α, which promote tumor progression. A novel approach involves vagus nerve stimulation to activate the cholinergic anti-inflammatory pathway via α7nAChR, reducing pro-inflammatory cytokines and potentially converting “cold” gliomas into “hot,” ICI-responsive tumors [271]. Concurrently, molecular targets such as TRAF7, which drives glioma recurrence by inhibiting senescence and cell cycle arrest, are being explored. Combining TRAF7 knockdown with lomustine enhances therapeutic efficacy by inducing senescence and G0/G1 arrest, mediated through KLF4 interaction [272]. Pediatric low-grade gliomas also exhibit RAS/MAPK-driven oncogene-induced senescence, offering a therapeutic window for targeted interventions [273]. Radiation therapy, while standard, paradoxically induces senescence in non-neoplastic brain cells such as astrocytes, which recruit myeloid cells and promote tumor regrowth via SASP-a process mitigated by senolytic agents [274]. Similarly, senescent brain macrophages contribute to immune dysfunction and glioma progression, highlighting their potential as prognostic markers or therapeutic targets [275]. In radiation-induced brain injury, pericyte senescence disrupts the blood-brain barrier and exacerbates glioma growth, but rapamycin-induced autophagy or senolytics such as all-trans retinoic acid can reverse these effects [276]. Epigenetic dysregulation in ATRX-deficient gliomas is another key focus, with SIRT2 inhibitors shown to induce senescence and impair tumor growth by remodeling chromatin and restoring KLF16-mediated transcriptional control [277]. Gambogic acid (GA) emerges as a promising therapy by targeting BMI1, a poly-comb group protein critical for glioma stem cell (GSC) self-renewal. GA binds BMI1’s RING domain, triggering its degradation and suppressing H2A ubiquitination, thereby inducing GSC apoptosis and enhancing temozolomide sensitivity [278]. Senescence-related gene signatures further stratify gliomas into prognostic subtypes, with CEBPB and LMNA linked to poor outcomes and immunosuppression, while dasatinib shows potential as a tailored therapy [279]. Together, these advances underscore the dual role of senescence in glioma suppression and progression, emphasizing the need for precision therapies targeting SASP, epigenetic regulators, and stemness pathways to improve patient outcomes.

### Hematologic system: blood cancer

Acute lymphoblastic leukemia (ALL), the most common pediatric cancer, is increasingly recognized for its long-term complications, including premature aging and cellular senescence in survivors. Studies reveal that diet quality significantly impacts immune aging, with the Healthy Diet Indicator positively correlating with thymic output markers such as T-cell receptor excision circles, while inflammation (elevated IL6 and CRP) exacerbates immune decline [280]. In philadelphia chromosome-like ALL, kinase-driven subtypes exhibit resistance to targeted therapies, but multi-omics profiling identifies a senescence-associated stem cell-like subpopulation reliant on MYC and AP1, which can be targeted with BCL2/JAKSTAT or SRC/ABL inhibitors [281]. Similarly, β-arrestin1 emerges as a tumor suppressor in T cell Acute Lymphoblastic Leukemia (TALL), inducing senescence via the RAS-p16-pRb-E2F1 pathway and improving chemotherapy response [282]. Chronic inflammation in ALL survivors, marked by elevated IL1β, IL6, and TNF-α, underscores accelerated aging, particularly in those treated before age 5 or with radiotherapy [283]. Precision therapies face challenges due to tumor heterogeneity; WEE1 inhibition in KMT2A-rearranged ALL diversifies cell states, with drug-tolerant populations reversible by BCR-signaling or metabolic inhibitors such as dasatinib and fatostatin [284]. The bone marrow TME plays a pivotal role, as leukemia cells induce senescence in mesenchymal stem cells (MSCs), reducing differentiation capacity and upregulating pro-leukemic factors such as IFI6, which activates the SDF1/CXCR4/ERK axis to fuel B-cell Acute Lymphoblastic Leukemia (BALL) progression [285]. Epigenetic dysregulation in TALL involves MYC-mediated control of TET1/TET2 enzymes, where TET1 promotes oncogenesis via ribosomal biogenesis, while TET2 acts as a tumor suppressor [286]. Bone marrow aging further complicates leukemia biology, with senescent MSCs creating a permissive niche for both pediatric BALL and adult AML, suggesting shared therapeutic targets [287, 288]. Paradoxically, TIS may initially suppress relapses, as shown in TALL mouse models, though long-term senescence contributes to secondary malignancies and frailty [289]. Collectively, these findings highlight the dual role of senescence in ALL, restraining relapses while driving late effects, and advocate for tailored strategies, including senolytics, epigenetic modulators, and microenvironment-targeted therapies, to improve outcomes for patients and survivors.

### Endocrine system: thyroid cancer

Thyroid cancer (TC) exhibits a complex relationship with cellular senescence, a process that can both inhibit and promote tumor progression depending on context. Anaplastic thyroid cancer (ATC), a highly aggressive form, frequently arises from the dedifferentiation of well-differentiated papillary or follicular TC, with type 2 deiodinase playing a crucial role in ATC proliferation and invasiveness by activating thyroid hormone thyroxine into triiodothyronine [290]. Senescence-related mechanisms are implicated in TC progression, as demonstrated by the downregulation of SESN2, a gene involved in oxidative stress response, in papillary thyroid carcinomas of patients aged ≥ 45, correlating with poorer prognosis [291]. Androgen receptor (AR) activation in TC cells induces senescence, marked by G1 arrest and increased expression of tumor suppressors such as p16 and p21, which may underlie the lower incidence of TC in men [292]. Senescence-related genes have been identified as predictive biomarkers for TC outcomes, with signatures involving ADAMTSL4, DOCK6, and FAM111B showing strong associations with immune infiltration and immunotherapy response [293, 294]. In PTC, recurrent genomic alterations, such as deletions on Chr22 and mutations in the RTKRAS pathway, contribute to early-stage tumor heterogeneity and progression, with senescence pathways often disrupted [295]. The BRAF inhibitor vemurafenib faces resistance in TC but combining it with Ref1 redox inhibitors can induce senescence and cell death by overloading autophagic flux [296]. Similarly, lenvatinib resistance in ATC can be overcome by CDK2 inhibition, which induces senescence via G1/S arrest [297]. Senescence also influences the TME, as senescent thyroid cells recruit and polarize M2-like macrophages, fostering a pro-tumoral environment [298, 299]. Aging exacerbates TC aggressiveness, with chromosomal alterations in loci 1p/1q and dysregulated proteostasis and ERK1/2 signaling driving poorer outcomes in patients aged ≥ 55 [300]. Additionally, obesity-related carcinogenesis, involving chronic inflammation and senescence, impacts TC pathogenesis, particularly in hormone-sensitive cancers [301]. TERT reactivation, through promoter mutations or epigenetic changes, avoids senescence and promotes TC proliferation [302]. Furthermore, ZNF24 induces TC cell senescence by inhibiting the Wnt/β-catenin pathway, offering a potential therapeutic target [303]. Senescence-related prognostic models, such as those involving E2F1 and SNAI1, effectively stratify TC patients by risk and immune infiltration patterns [304]. Ubiquitin-specific protease 7 also emerges as a biomarker, with its expression linked to DNA repair and senescence pathways in TC [305]. Collectively, these studies highlight senescence as a double-edged sword in TC, influencing tumor behavior, therapeutic response, and patient prognosis, while offering novel targets for precision medicine.

### Integumentary system: skin cancer

Skin cancer (SKC), including melanoma and nonmelanoma variants, is intricately linked to cellular senescence, a process that serves as both a tumor-suppressive mechanism and a contributor to therapy resistance and tumor progression. Ultraviolet radiation, a primary risk factor for SKC, induces senescence through DNA damage, oxidative stress, and mitochondrial dysfunction, while also promoting a pro-inflammatory SASP that can paradoxically foster tumorigenesis [306–308]. In melanoma, familial mutations in CDKN2A, RB1, and TERT attenuate senescence, enabling uncontrolled proliferation [309], whereas ERK5 inhibition triggers senescence via LTBP1-mediated TGF-β1 activation, which suppresses tumor growth and enhances immunotherapy response [310]. Senescence also plays a dual role in therapy resistance: genotoxic therapies such as carboplatin/paclitaxel induce senescence in melanoma cells, rendering them susceptible to Bcl2 inhibitors, while targeted BRAF/MEK inhibition produces senescent-like cells resistant to such treatments [311]. Similarly, chemotherapy-induced senescent melanoma cells upregulate ITGA1, a potential biomarker for targeted senolytic therapy [312]. In Merkel cell carcinoma, viral oncoprotein MCPyV-LT induces p21-dependent senescence to maintain viral persistence, suggesting senolytics such as navitoclax as preventive strategies [313]. The TME is shaped by senescent fibroblasts and CAFs, which promote SKC progression via lamin A/C phosphorylation and ANKRD1-driven myofibroblast activation, independent of senescence [314, 315]. UV-induced photoaging further exacerbates immunosensecence, impairing T-cell function and dendritic cell activity, which ALAPDT reverses by rejuvenating immune responses [316]. Natural compounds such as Withania somnifera (Ashwagandha) and aloe-derived exosomes (ADNPs) mitigate senescence by reducing oxidative stress, restoring extracellular matrix homeostasis, and activating Nrf2/ARE pathways [317, 318]. Epigenetic modulators, such as telomerase activators, suppress melanoma growth by downregulating hTERT and upregulating HDACs [319], while acetyl zingerone combats photoaging by neutralizing ROS and stabilizing collagen [320]. Laser therapies prevent keratinocyte carcinoma by removing DNA-damaged cells and reducing fibroblast senescence [321]. Chronic inflammation from SASP exacerbates immune aging, linking senescence to type 2 inflammatory disorders and pigmentation defects such as vitiligo [308]. The anti-aging formula delays skin senescence by targeting CXCR2 to inhibit p38/p53 signaling [322]. Despite its protective role, senescence accumulation in aged skin creates a permissive milieu for cancer, as seen in D2-driven anaplastic TC models, where senescence evasion fuels proliferation [290]. Collectively, senescence in SKC represents a double-edged sword, necessitating context-specific strategies, such as senolytics to eliminate harmful senescent cells or SASP modulators to disrupt protumor signaling, to improve therapeutic outcomes [323, 324].

## Therapeutic targeting of senescence in cancers

**Fig. 6:**
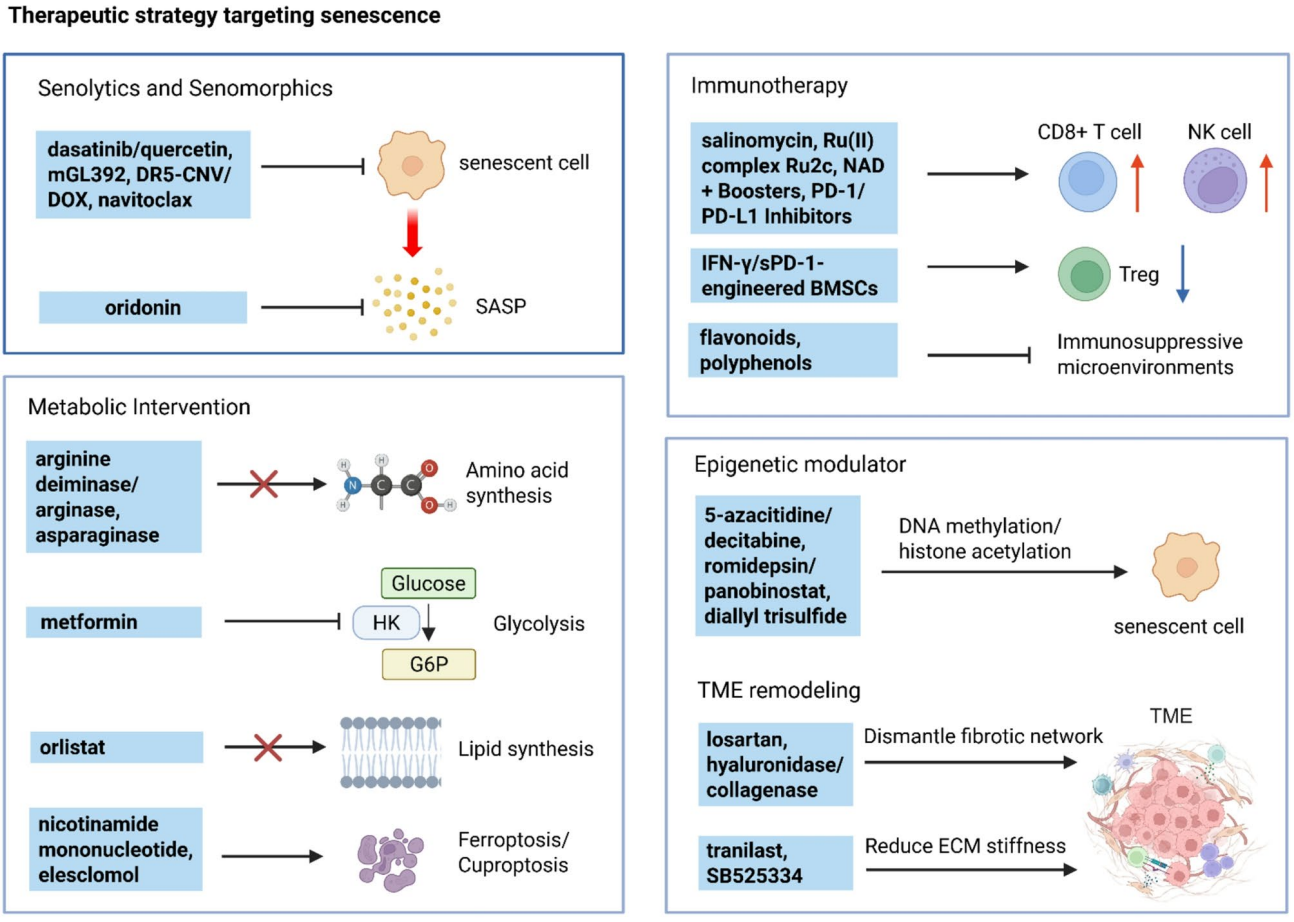
Summary of main therapeutic strategy targeting senescence. Senolytics and senomorphics (e.g., dasatinib/quercetin, mGL392, DR5-CNV/DOX, navitoclax) directly eliminate senescent cells or suppress their harmful SASP secretions. Metabolic interventions (e.g., arginine deiminase/arginase, asparaginase, metformin, orlistat, nicotinamide mononucleotide, elesclomol) disrupt amino acid synthesis, glycolysis, and lipid metabolism and induce apoptosis. Immunotherapies (e.g., salinomycin, Ru(II) complex Ru2c, NAD + Boosters, PD-1/PD-L1 Inhibitors, IFN-γ/sPD-1-engineered BMSCs, flavonoids, polyphenols) enhance immune clearance of senescent cells. Epigenetic modulators (e.g., 5-azacitidine/decitabine, romidepsin/panobinostat, diallyl trisulfide) reprogram senescent cells via DNA methylation or histone acetylation. Lastly, TME remodeling agents (e.g., losartan, hyaluronidase/collagenase, tranilast, SB525334) modify TME to improve therapeutic access

Cellular senescence plays a dual role in cancer acting as a tumor suppressor by halting damaged cell proliferation while promoting malignancy through the pro-inflammatory SASP. This paradox has spurred innovative therapeutic strategies, including senolytics to eliminate senescent cells and senomorphics to modulate SASP. Advances in nanotherapy, immunotherapy, metabolic interventions, and epigenetic modulators aim to exploit senescence vulnerabilities while mitigating its pro-tumor effects (Fig. 6 and Table 2).

**Table 2:** Therapeutic targeting of senescence in different cancer types

Drug Name	Targeted cancer types	Strategy	Effects	References
Dasatinib/quercetin	Melanoma	Senolytics	Selectively eliminates senescent cells; reduces tumor burden.	[328, 329]
mGL392	Melanoma	Senolytic nanotherapy	Lipofuscin-targeted dasatinib conjugate; enhances specificity and reduces toxicity.	[328, 329]
Oridonin	Gastric cancer	Senomorphics	Suppresses SASP (reduces IL6/IL8 by 55–62%); preserves senescence markers.	[330]
DR5-CNV/DOX	Gastric cancer	Senolytics	Eliminates senescent tumor cells; overcomes SASP-induced immune suppression.	[331]
Navitoclax	Vestibular schwannomas	Senolytics	Clears chemotherapy-induced senescent cells.	[334, 335]
Salinomycin	Gastric cancer	Immunotherapy	Enhances NK/CD8 + T-cell clearance of senescent cells via IL-18 release.	[333]
Flavonoids	Gastric cancer	Immunotherapy	Reverse immunosuppressive microenvironments; enhance PD-1/PD-L1 efficacy.	[28]
Polyphenols	Gastric cancer	Immunotherapy	Mitigate immunosenescence; synergize with checkpoint inhibitors.	[28]
PD-1/PD-L1 Inhibitors	Non-small cell lung cancer, Gastric cancer, Colorectal cancer	Immunotherapy	Reverses T-cell exhaustion; synergizes with senolytics.	[332, 336, 362]
Ru(II) complex Ru2c	Colorectal cancer	Immunotherapy	Triggers apoptosis-ferroptosis-senescence synergy; activates CD8 + T cells.	[337]
IFN-γ/sPD-1-engineered BMSCs	Lung adenocarcinoma	Immunotherapy	Reduces Tregs by 40%; induces tumor senescence via p16 upregulation.	[269]
NAD + Boosters	Preclinical (Aged T cells)	Immunotherapy	Reverses T-cell senescence; restores OXPHOS capacity.	[58]
Arginine deiminase/arginase	Pancreatic cancer, colorectal cancer, Hepatocellular carcinoma	Metabolic interventions	Starves ASS1-deficient tumors of arginine.	[341, 342]
Asparaginase	Leukemia	Metabolic interventions	Depletes asparagine; limited by ASNS upregulation.	[343]
Metformin	HPV + Cervical cancer	Metabolic interventions	Inhibits HK2; shifts cells to mitochondrial metabolism.	[343]
Orlistat	Prostate cancer	Metabolic interventions	Inhibits FASN; induces apoptosis.	[344]
Nicotinamide mononucleotide	Preclinical models	Metabolic interventions	Boosts NAD+; induces autophagy/ferroptosis.	[345]
Elesclomol	Therapy-resistant cancers	Metabolic interventions	Induces oxidative stress and cuproptosis; targets mitochondrial-rich tumors.	[346]
5-azacitidine/decitabine	Prostate and colorectal Cancers	Epigenetic modulators	Reactivates p16 INK4a/p21 via DNA demethylation.	[347, 348]
Romidepsin/panobinostat	Breast and ovarian cancers	Epigenetic modulators	Upregulates p21; modulates SASP.	[349, 350]
Diallyl trisulfide	Breast and Gastric cancers	Epigenetic modulators	Demethylates DNA/acetylates histones; induces senescence.	[354–356]
losartan	Pancreatic cancer	TME remodeling	Reduces fibrosis; improves drug delivery.	[361]
Galunisertib	Colorectal cancer	TME remodeling	Synergizes with PD-L1 inhibitors.	[362, 363]
SB525334	Pancreatic cancer	TME remodeling	Breaks down myCAF barriers with docetaxel micelles.	[364]
Tranilast	Breast cancer	TME remodeling	Reduces ECM stiffness; enhances anti-PD-1 efficacy.	[365]
Hyaluronidase/collagenase	Breast cancer	TME remodeling	Dismantles fibrotic networks.	[365]
²²³Ra/Ba single-atom Nanozymes	Broad (SASP-driven tumors)	Senolytics + Radiotherapy	Induce senescence via catalase/peroxidase-mimicry; synergize with anti-PD-L1 to eliminate immunosuppression.	[332]
M7824	Colorectal cancer	Immunotherapy + TME remodeling	Enhances CD8 + T-cell infiltration.	[362, 363]

*Abbreviations*: *BMSCs* Bone Marrow Stromal Cells, *SASP* Senescence-Associated Secretory Phenotype, *TME* Tumor Microenvironment, *myCAF* Myofibroblastic Cancer-Associated Fibroblasts, *ECM* Extracellular Matrix, *Tregs* Regulatory T cells, *OXPHOS* Oxidative Phosphorylation

### Senolytics and senomorphics

Cellular senescence, a state of irreversible cell cycle arrest, plays a paradoxical role in human health, acting as a tumor suppressor by halting damaged cell proliferation while promoting age-related pathologies through chronic inflammation via the SASP [325, 326]. SASP comprises pro-inflammatory cytokines (IL6, IL8), chemokines, and proteases that remodel tissue microenvironments, fostering conditions conducive to cancer progression, particularly in bone niches where chronic inflammation drives tumorigenesis [327]. This duality has spurred the development of senotherapeutics, including senolytics that selectively eliminate senescent cells, and senomorphics that suppress SASP. Recent innovations such as mGL392-a lipofuscin-targeted nanotherapy conjugating dasatinib to a lipofuscin, binding scaffold, demonstrate enhanced specificity, reducing melanoma tumor burden in mice while minimizing systemic toxicity compared to conventional senolytics [328, 329]. In bladder dysfunction, polyploid umbrella cells exhibit senescence markers (elevated cyclin D1) resistant to senolytics, suggesting tissue-specific senescence roles in maintaining urothelial barrier integrity. Similarly, GC immunotherapy faces challenges due to immune senescence, but natural senotherapeutics such as flavonoids and polyphenols show promise in reversing immunosuppressive microenvironments and enhancing PD1/PD-L1 inhibitor efficacy [28]. Oridonin, a diterpenoid from *Isodon* plants, selectively inhibits SASP (reducing IL6/IL8 by 55–62%) in senescent cells by suppressing NF-κB and p38 pathways while preserving senescence markers (SA-β-gal, p21). This senomorphic activity makes it a promising therapeutic candidate for SASP-driven pathologies such as chronic inflammation and cancer, without reversing growth arrest [330]. Senolytics such as DR5CNV and DOX can eliminate senescent tumor cells, which promote SASP-induced immune suppression [331]. Combination therapies such as anti-PD-L1 + senolytics may exploit SASP to prime immune responses while preventing chronic inflammation [332]. CRISPR screens identified SLC25A23 as a senescent cell vulnerability; its inhibition disrupts calcium homeostasis, triggering PANoptosis through ROS/JNK/DR5 activation, while combining DR5 agonists with salinomycin enhances NK/CD8 + T cell-mediated clearance of senescent GC cells through IL18 release [333]. In vestibular schwannomas, chemotherapy-induced senescence (evidenced by p21 upregulation and SASP activation) sensitizes tumors to navitoclax, a Bcl2 inhibitor, highlighting a “one-two punch” strategy: pro-senescence therapy followed by senolysis [334]. Nanomedicine advances include galactose-functionalized micelles that selectively deliver navitoclax to senescent cells via β-galactosidase activation, improving therapeutic indices [335]. Radiotherapeutic approaches leveraging (223) Ra/Ba single-atom nanozymes further exemplify this strategy; these agents induce senescence through catalase/peroxidase-mimicry, then synergize with anti-PD-L1 to eliminate SASP-mediated immunosuppression [332]. Despite progress, challenges persist in optimizing senolytic specificity, understanding tissue-specific senescence responses, and mitigating TIS in residual cancer cells.

### Immunotherapy

The interplay between cellular senescence and immune dysfunction represents a critical frontier in cancer immunotherapy, with profound implications for treatment durability and patient outcomes. T cell exhaustion and senescence emerge as parallel yet distinct barriers to sustained anti-tumor immunity-exhaustion arising from chronic antigen exposure in immunosuppressive microenvironments (reversible via checkpoint blockade), while senescence reflects irreversible proliferative arrest driven by aging or stress [336]. In NSCLC, Single-cell RNA sequencing reveals T cell senescence correlates with poor prognosis, with a validated risk model (SLC2A1/TNS4/GGTLC1 signature, AUC > 0.8) predicting immunotherapy resistance. Mechanistically, senescent T cells exhibit upregulated glycolysis via SLC2A1/GLUT1 and SASP secretion (IL6/IL8), fostering tumor permissive microenvironments [263]. Similar dynamics occur in GC, where TXNIP-mediated proliferation influences immune evasion, with high-risk patients showing elevated stromal scores and PD-L1 expression but paradoxically poorer response to ICIs due to Treg infiltration [111].

Emerging strategies target these barriers through multi-modal approaches: Metabolic reprogramming: NAD + boosters reverse age-related T cell dysfunction, synergizing with PD1 blockade to restore OXPHOS capacity in preclinical models [58]. Gene-modified cell therapy: IFNγ/sPD1-engineered BMSCs target lung adenocarcinomas, simultaneously suppressing PI3K/AKT, reducing Tregs by 40%, and inducing tumor senescence via p16 upregulation [269]. Notably, epigenetic modifications such as RNA pseudouridylation (Ψ) link senescence to CRC progression, where low Ψ expression correlates with mTOR/TGF-β-driven SASP and predicts immunotherapy response via a novel “Psi Score” [105]. Similarly, the Ru(II) complex Ru2c exemplifies innovative senoimmunotherapy, inhibiting BRD4 at nanomolar affinity to trigger apoptosis-ferroptosis-senescence synergy (51-fold potency over JQ1) while activating CD8 + T cells via immunogenic DAMPs [337].

Collectively, these findings illuminate senescence as a double-edged sword, targetable via senolytics, senomorphics, or metabolic modulators, but requiring precision to avoid exacerbating immune dysfunction. Future directions include optimizing tissue-specific delivery such as prostate-specific membrane antigen-guided radioligands, exploiting CRISPR-engineered CART cells resistant to senescence, and integrating single-cell ATACseq/spatial transcriptomics to decode microenvironmental crosstalk [338–340].

### Metabolic interventions

Metabolic interventions are a promising strategy to target cancer cell senescence, focusing on disrupting the unique metabolic dependencies of tumors to inhibit their growth and survival. A key approach involves amino acid deprivation, particularly arginine, which is crucial for protein synthesis and immune function. Many cancers, such as pancreatic, colorectal, and HCC, exhibit downregulated argininosuccinate synthetase, rendering them auxotrophic for arginine [341]. Enzymes such as arginine deiminase and arginase are employed to deplete extracellular arginine, selectively starving these tumors while sparing normal cells [342]. Asparagine deprivation exploits the semi-essential nature of this amino acid in cancers such as leukemia. While asparaginase shows therapeutic success, compensatory upregulation of asparagine synthase limits its efficacy, underscoring the need for combinatorial approaches. Another metabolic intervention lies in glycolysis, where targeting hexokinase 2 (HK2), the rate-limiting enzyme, alters energy metabolism in cancers such as HPV + CC [343]. Inhibition of HK2 via shRNA or metformin shifts cells away from glycolysis, enhances mitochondrial function, and sensitizes tumors to irradiation by inducing apoptosis and suppressing oncogenic HPV16 E7-driven metabolic reprogramming. Similarly, lipid metabolism is disrupted using Orlistat, an FDA-approved obesity drug repurposed to inhibit fatty acid synthase (FASN), thereby halting tumor proliferation and inducing apoptosis in PCa models [344]. Nicotinamide mononucleotide elevates NAD + levels, activates AMPK/mTOR signaling, and induces autophagy and ferroptosis, effectively suppressing tumor growth in preclinical studies [345]. Finally, the mitochondrial metabolism-targeting drug Elesclomol is discussed for its dual role in inducing oxidative stress and cuproptosis, a copper-dependent cell death mechanism, particularly in cancers with heightened mitochondrial activity, such as therapy-resistant or glycolytic-inhibited tumors. Despite initial clinical setbacks, Elesclomol’s specificity for mitochondrialrich cancers and potential synergy with platinum drugs or proteasome inhibitors offers renewed therapeutic promise [346].

Collectively, these metabolic interventions exploit cancer-specific vulnerabilities, from nutrient auxotrophy to altered energy pathways, but face challenges such as metabolic plasticity and resistance.

### Epigenetic modulators

Epigenetic modulators represent a promising therapeutic strategy for treating cancers by inducing senescence, leveraging the reversible nature of epigenetic alterations to halt tumor progression and restore tumor suppressor functions. DNA methyltransferase inhibitors such as 5-azacitidine and decitabine demethylate silenced promoters, reactivating p16 INK4a and p21 in prostate and colorectal cancers, as evidenced by DNMT1 downregulation and TET1/TET2 restoration [347, 348]. Histone deacetylase inhibitors (HDACis) such as romidepsin and panobinostat enhance histone acetylation, promoting senescence in BC and OC by upregulating p21 and suppressing cyclin-dependent kinases, while also modulating the SASP to remodel the tumor microenvironment [349, 350]. For instance, HDACis combined with PD1 inhibitors in mismatch repair-proficient CRC-induced immune infiltration and senescence, albeit with limited clinical efficacy, highlighting the need for optimized epigenetic-immune combinations [351, 352]. Dual-targeting agents, such as compounds 22a and 22b, synergistically suppress proliferation in hematological malignancies by concurrently inhibiting poly-comb repressive complex 2 and HDACs, leading to RB1-mediated senescence and apoptosis [353]. Natural compounds, including polyphenols and garlic-derived diallyl trisulfide, exert epigenetic effects by demethylating DNA or acetylating histones, inducing senescence in BC and GC while mitigating oxidative stress [354–356]. Furthermore, PROTAC-based degraders mimic CRISPR-induced knockdowns, enhancing senescence induction by destabilizing epigenetic regulators critical for cancer cell survival [357]. Epigenetic reprogramming via nanocarriers, such as self-assembled DNMT inhibitors/HDACis nanofibers, improves drug retention and specificity, effectively inducing senescence in GC by coordinately reversing aberrant DNA methylation and histone deacetylation [348]. Senescence induction is also facilitated by modulating chromatin architecture; lamin A and histone H3 interactions disrupted by H3.3K27M mutations or HDACis lead to nuclear morphology changes and senescence-associated heterochromatin foci [358]. However, challenges persist, including off-target effects and transient responses, as seen in mismatch repair proficient CRC trials [351]. Despite these limitations, epigenetic modulators-through their ability to reprogram senescence pathways and synergize with immunotherapy or chemotherapy-offer a transformative approach to cancer treatment, particularly in malignancies with dysregulated epigenetic landscapes [359, 360].

### Microenvironment remodeling

The TME plays a pivotal role in cancer progression and therapy resistance, making its remodeling a promising therapeutic strategy. A key approach involves targeting TGF-β signaling, a major driver of fibrosis, immunosuppression, and metastasis. TGF-β inhibitors such as losartan, galunisertib, and SB525334 disrupt CAF activation, reduce ECM deposition, and restore immune cell function. For instance, losartan normalizes the TME in PC by decreasing fibrosis and improving drug delivery [361], while galunisertib synergizes with M7824 in CRC to enhance CD8 + T-cell infiltration and suppress tumor growth [362, 363]. Similarly, SB525334 combined with docetaxel micelles breaks down my-CAF barriers in PC, facilitating drug penetration and reducing metastasis [364]. ECM-targeting agents such as hyaluronidase and collagenase further dismantle fibrotic networks, as demonstrated in BC models where tranilast (a TGF-β inhibitor) combined with Doxil reduces ECM stiffness, improves vessel perfusion, and boosts anti-PD1 efficacy [365]. These strategies also address immune evasion: TGF-β blockade reprograms immunosuppressive TANs (N2) into anti-tumor N1 phenotypes, enhancing radiotherapy responses [366], while EMILIN1 + CAFs in BC counteract TGF-β to recruit cytotoxic T cells [367]. Additionally, novel delivery systems such as neutrophil micropharmacies co-deliver galunisertib and rinotecan to CRC tumors, achieving > 4-fold higher drug accumulation and sensitizing tumors to immune checkpoint blockade [363]. Probiotic-based approaches, such as EcN@(CSSA)_2_ microgels, further enhance TGF-β inhibition, promoting CD8 + T cell infiltration and immunogenic cell death [368]. IMSN-PEG-TI combines TGF-β inhibition with catalytic ROS generation, polarizing M2 macrophages to M1 and regenerating H_2_O_2_ for tumor killing [369]. However, challenges remain due to CAF heterogeneity, IFNγ-iCAFs paradoxically suppress tumors via EMILIN1, and the risk of therapy-induced metastasis. Despite these complexities, TME remodeling strategies, particularly those combining TGF-β inhibition with immunotherapy or nanomedicine [366], offer transformative potential for overcoming resistance and improving outcomes in aggressive cancers.

## Challenges

### Off-target effects

Senolytic drugs, designed to eliminate senescent cells, hold promise for treating aging and cancer by targeting pro-inflammatory SASP components [327]. However, their non-specificity raises concerns about the unintended depletion of anti-tumor senescent cells, which play critical roles in tumor suppression and tissue homeostasis. Current senolytics, such as dasatinib/quercetin, often lack precision. To address this, researchers have engineered targeted delivery systems. For example, galactose-functionalized micelles exploit elevated lysosomal β-galactosidase in senescent cells to deliver the Bcl2 inhibitor navitoclax with reduced toxicity to healthy cells [335]. Similarly, mGL392, a lipofuscin-binding senolytic platform conjugated to dasatinib, is invented to target lipofuscin of senescent cells in melanoma models while minimizing systemic side effects [370]. Another innovative approach involves iron oxide nanoparticles (MNP@CD26@17D) functionalized with an anti-CD26 antibody and loaded with the HSP90 inhibitor 17DMAG [371]. These nanoplatforms induce dual apoptosis and ferroptosis in senescent fibroblasts and squamous carcinoma cells. While newer platforms improve specificity by targeting senescent cells, off-target effects persist. For instance, increased lipofuscin and SA-β-gal activity are not specific to senescence, and their use results in false-positive outcomes. Importantly, CD26 is also expressed on T-cells, and MNP@CD26@17D potentially causes immunosuppression. Future strategies should focus on integrating cell type-specific markers and combinatorial approaches to preserve anti-importer functions and minimize off-target effects [332]. Additionally, standardized detection methods and tissue-specific senolytic delivery systems are essential for minimizing these risks.

### Biomarkers limitation

Targeting cellular senescence in cancer therapy requires reliable biomarkers to distinguish pro-tumorigenic from antitumorigenic senescent cells. Current challenges stem from the heterogeneity of senescent phenotypes and the lack of consensus markers [372].

The tumor suppressor p16 INK4a, a canonical cyclin-dependent kinase inhibitor, serves as a well-established biomarker of cellular senescence and is critically involved in both aging processes and tumor suppression pathways. In CRC, p16 INK4a expression in peripheral immune cells (CD45+, CD3+, CD14+) showed 78% sensitivity and 71% specificity as a diagnostic tool [373]. However, p16 INK4a’s utility is limited by its variable expression across tissues and its presence in both tumor-suppressive and pro-tumorigenic contexts [374]. Despite its widespread use as a senescence indicator, SA-β-gal’s enzymatic activity lacks cellular specificity. New flow cytometry-based methods now enable simultaneous quantification of SA-β-gal with p16 INK4a and γ-H2AX, improving specificity [372]. The SASP includes cytokines and proteases that promote tumor progression. In HER2 + BC, senescent cells exclusively secreted IL6, driving tumor growth [375]. However, SASP factors are highly context-dependent, with both pro and anti-tumor effects, complicating their use as universal biomarkers. A multi-parametric approach combining p16 INK4a, SA-β-gal, and SASP profiling is essential to address senescence heterogeneity. Standardized detection protocols and tissue-specific validation are critical for clinical translation [372].

### Heterogeneity of senescent cells

The heterogeneity of senescent cells poses a significant challenge for cancer therapy due to their diverse roles in tumor progression, resistance mechanisms, and immune modulation. Senescent cells exhibit context-dependent behaviors, functioning as both tumor suppressors and promoters, which complicates therapeutic targeting [376]. For instance, in BLCA, senescent umbrella cells contribute to barrier integrity but resist senolytic treatments such as dasatinib and quercetin, highlighting tissue-specific resistance [27]. Similarly, GC studies reveal that autophagy and senescence-related gene signatures stratify patients into distinct prognostic clusters, with high-risk groups showing immune evasion and poor responses to chemotherapy [111]. In BC, senescence escape mechanisms, such as galectin-8 upregulation drive multi-drug resistance [377], while TIS in HCC exposes immunotherapeutic vulnerabilities but also induces heterogeneous surface antigens such as CD95 and CD276, complicating targeted approaches [378]. Circulating tumor cells further mirror this heterogeneity, reflecting dynamic senescence states that evade detection and treatment [379]. Single-cell analyses underscore the spatial and temporal diversity of senescent cells within the TME, where they influence immune interactions and therapeutic outcomes [380]. Metabolic reprogramming, such as the LDHA-NDRG1 axis in HCC, links lactate-driven epigenetic modifications to senescence evasion, creating resistant subpopulations [123]. Telomere and senescence-related gene signatures in BC reveal divergent fibroblast and immune cell subpopulations with varying therapeutic sensitivities [165], while in GBM, senescence-related genes such as SOCS1 and PHB2 stratify patients by risk but also correlate with immune infiltration and drug resistance [381]. SASP remodels TME, promoting inflammation, fibrosis, and immunosuppression, which can either inhibit or accelerate tumor growth [376, 382]. Hypoxia-induced senescent fibroblasts in ESCC secrete IGF1 to promote stemness and chemoresistance [382], and in PC, senescence-driven heterogeneity correlates with poor prognosis and altered immune checkpoints [383]. Bone metastases in NSCLC exhibit a senescent TME with dysfunctional CD4Tstr cells, fostering an immunocompromised niche [384]. Collectively, the heterogeneity of senescent cells-spanning metabolic, epigenetic, and immune mechanisms-demands tailored approaches to overcome resistance and improve therapeutic efficacy.

### Delivery and Pharmacokinetic barriers

The effective delivery of senescence-targeting agents faces formidable pharmacological hurdles that limit their clinical translation. These challenges stem from both the unique properties of senescent cells and the pathological features of the tumor microenvironment.

Physical barriers in the TME present the first major obstacle. Dense fibrotic stroma, particularly in cancers such as pancreatic and breast, severely restricts drug penetration [385]. Senescent CAFs exacerbate this through excessive ECM deposition including collagen, hyaluronic acid, and fibronectin, creating high interstitial fluid pressure that limits convective transport [386]. Even when drugs penetrate the tumor core, senescent cells often reside in specialized niches such as perivascular areas or tumor margins that are poorly accessible [387, 388]. This spatial heterogeneity means that drugs may reach proliferating tumor cells while bypassing senescent populations.

The pharmacological properties of many senescence-targeting agents further complicate delivery. Most senolytics and senomorphics are small molecules with unfavorable pharmacokinetics rapid clearance, poor solubility, and limited tumor accumulation [389, 390]. Epigenetic modulators such as HDAC inhibitors suffer from similar issues, with short half-lives requiring frequent dosing that increases toxicity [391, 392]. The hydrophobic nature of many TGF-β inhibitors such as galunisertib also challenges formulation for systemic delivery [393]. Senescence therapy delivery faces TME barriers, poor drug penetration, and unfavorable pharmacokinetics, limiting clinical translation.

## Future directions

scRNAseq is revolutionizing the understanding of senescent cell heterogeneity in tumors, revealing context-dependent roles in cancer progression and therapy resistance. Recent studies highlight its potential to dissect the complex TME, where senescent cells exhibit divergent functions, from promoting wound healing to driving immunosuppression [394, 395]. scRNAseq in GBM and PC uncovered distinct senescence-associated gene signatures (CCDC151, CAV1) linked to prognosis [396, 397]. For example, CAV1 + senescent cells in PC promote metastasis, while MDH1 + senescent BC cells modulate drug sensitivity [397, 398]. In HCC, scRNAseq revealed senescent cells remodel the TME via CREM-mediated extracellular matrix interactions, correlating with poor immunotherapy response [399]. Similarly, BLCA studies identified LIG1 + senescent cells driving EMT and immune evasion [245]. Integrating scRNAseq with ELASTomics can pinpoint senescence-specific surface markers such as RRAD for precision senolysis [400].

Epigenetic clocks, which measure biological age through DNA methylation patterns, are emerging as powerful tools for developing personalized senotherapies in cancer. Studies reveal that tumors exhibit distinct epigenetic aging profiles, such as accelerated aging in gliomas and PCa [401, 402]. These clocks can identify high-risk patients, such as those with Hannum’s EEAA (extrinsic epigenetic age acceleration), which predicts a 6% increased cancer risk per year post-cerebrovascular events (HR = 1.06, *p* = 0.002) [403]. Epigenetic entropy in BC distinguishes aggressive “young” tumors (high proliferation) from stable “old” tumors (immune-rich), guiding early intervention [404]. Exercise reduced biological age in BC survivors (ELOVL2 clock), suggesting lifestyle interventions may complement senotherapies [405]. Integrating multi-clock models with senolytic regimens could optimize precision oncology, while addressing platform disparities.

Emerging research highlights the gut microbiome as a key modulator of cellular senescence, offering novel avenues for cancer therapy. The microbiome influences senescence through multiple mechanisms. Beneficial gut bacteria produce short-chain fatty acids such as butyrate, which reduce oxidative stress and inflammation, thereby delaying senescence. Conversely, *Pseudomonas aeruginosa* accelerates senescence via DNA damage and ROS production [406]. Fecal microbiota transplant from long-lived Ames dwarf mice to normal mice reprogrammed the gut microbiome, downregulating senescence markers (p21) and improving metabolic health, which suggests microbiome transplantation could mitigate senescence-associated cancer progression [407]. Dasatinib/quercetin modulates the microbiome in inflammatory bowel disease, reducing pro-inflammatory bacteria and enhancing anti-inflammatory species [408]. Similarly, *Castanea crenata* flower extract restored autophagy and polyamine levels, metabolites produced by the microbiome, delaying muscle senescence [409]. Together, single-cell multi-omics, epigenetic clocks, and microbiome modulation are transforming our understanding of senescence heterogeneity in cancer, enabling precision senotherapies that target context-dependent pro-tumorigenic mechanisms while preserving beneficial functions.

## Conclusion

Cellular senescence embodies a profound duality in cancer biology, serving as both a tumor-suppressive mechanism and an unexpected catalyst for malignancy. In its acute phase, senescence acts as a critical barrier against tumorigenesis by enforcing stable cell cycle arrest through OIS and DDR pathways. However, when senescence becomes chronic, SASP and immunosenescence foster a permissive TME characterized by chronic inflammation, immune dysfunction, and therapeutic resistance, key hallmarks of aggressive cancers.

Therapeutic strategies targeting senescence are rapidly evolving. Dasatinib/quercetin combinations and JAK/STAT inhibitors demonstrate clinical potential by selectively eliminating senescent cells or suppressing harmful SASP components, respectively. Complementary approaches, including arginine depletion and HDAC inhibitors, further expand the arsenal against senescence-driven malignancy. Yet significant challenges remain: the risk of off-target effects on vital non-senescent cells, the lack of universal biomarkers to distinguish protective versus pathological senescence, and the striking heterogeneity of senescent cell populations across tumor types and stages.

Emerging technologies are poised to overcome these limitations. Single-cell multi-omics platforms can decode senescence heterogeneity with unprecedented resolution, while epigenetic clocks may predict therapy response based on a tumor’s “senescence age”. Microbiome engineering, through probiotics or dietary interventions, offers a novel avenue to modulate systemic senescence. Critically, the success of these strategies hinges on understanding the spatiotemporal dynamics of senescence within the TME.

As we advance, the ultimate goal is clear: to develop precision therapies that selectively harness senescence’s protective functions while neutralizing its tumor-promoting effects. Achieving this balance will not only improve cancer treatment but also illuminate fundamental connections between aging, cellular stress responses, and malignancy. The road ahead demands interdisciplinary collaboration, but the potential to transform cancer care by targeting senescence is undeniable.

## Data Availability

No datasets were generated or analysed during the current study.

## Abbreviations

AC: Acetyl-CoA
ACC: Acetyl-CoA Carboxylase
ACLY: ATP Citrate Lyase
AhR: Aryl Hydrocarbon Receptor
AKT: Protein Kinase B
ALL: Acute Lymphoblastic Leukemia
AMPK: AMP-Activated Protein Kinase
AR: Androgen Receptor
ASCT2: Alanine-Serine-Cysteine Transporter 2
ATM: Ataxia Telangiectasia Mutated
ATR: Ataxia Telangiectasia and Rad3-Related
ATC: Anaplastic Thyroid Cancer
BALL: B-cell Acute Lymphoblastic Leukemia
BC: Breast cancer
BLCA: Bladder cancer
BRCA1: Breast Cancer Gene 1
CAFs: Cancer-Associated Fibroblasts
cGAS-STING: Cyclic GMP-AMP Synthase-Stimulator of Interferon Genes
CDK: Cyclin-Dependent Kinase
CDK2/4/6: Cyclin-Dependent Kinase 2/4/6
CHK1/2: Checkpoint Kinase 1/2
CRC: Colorectal Cancer
Cyclin D/E: Cyclin D/E (Cell Cycle Regulators)
DDR: DNA Damage Response
DNMT1: DNA Methyltransferase 1
dNTP: Deoxynucleotide Triphosphate
EC: Endometrial Cancer
ECM: Extracellular Matrix
MCT4: Monocarboxylate Transporter 4
E2F: E2F Transcription Factor
EGF: Epidermal Growth Factor
EMT: Epithelial-Mesenchymal Transition
ESCA: Esophageal cancer
ERK: Extracellular Signal-Regulated Kinase
FASN: Fatty Acid Synthase
FGF: Fibroblast Growth Factor
G6P: Glucose-6-Phosphate
GBM: Glioblastoma
GC: Gastric Cancer
GLUT1: Glucose Transporter 1
GLS1: Glutaminase 1
GRB2: Growth Factor Receptor-Bound Protein 2
HCC: Hepatocellular Carcinoma
HDAC: Histone Deacetylase
HIF: Hypoxia-Inducible Factor
HK2: Hexokinase 2
IB: Inhibitor of Kappa B
ICI: Immune Checkpoint Inhibitor
IDO: Indoleamine 2,3-Dioxygenase
IKK: Inhibitor of Nuclear Factor Kappa-B Kinase
IL-6: Interleukin-6
IRS: Insulin Receptor Substrate
LDHA: Lactate Dehydrogenase A
lncRNA: Long Non-Coding RNA
MCT4: Monocarboxylate Transporter 4
MDSCs: Myeloid-Derived Suppressor Cells
MEK: Mitogen-Activated Protein Kinase Kinase
MHC-II: Major Histocompatibility Complex Class II
MMR: Mismatch Repair
mTOR: Mechanistic Target of Rapamycin
NAD+: Nicotinamide Adenine Dinucleotide
NF-κB: Nuclear Factor Kappa B
NFATc1: Nuclear Factor of Activated T-Cells 1
NK cells: Natural Killer cells
NSCLC: Non-Small Cell Lung Cancer
OIS: Oncogene-Induced Senescence
OC: Ovarian Cancer
OXPHOS: Oxidative Phosphorylation
PAICS: Phosphoribosylaminoimidazole Carboxylase and Succinyltransferase
p16: Cyclin-Dependent Kinase Inhibitor 2 A
p21: Cyclin-Dependent Kinase Inhibitor 1 A
p53: Tumor Protein p53
PARP: Poly (ADP-Ribose) Polymerase
PCa: Prostate Cancer
PD-1: Programmed Cell Death Protein 1
PDGF: Platelet-Derived Growth Factor
PDH: Pyruvate Dehydrogenase
PDL1: Programmed Death-Ligand 1
PEP: Phosphoenolpyruvate
PI3K: Phosphoinositide 3-Kinase
PIP2/PIP3: Phosphatidylinositol (4,5)-Bisphosphate/Trisphosphate
PKM2: Pyruvate Kinase M2
RAS-GDP/GTP: RAS Guanosine Diphosphate/Triphosphate
RB: Retinoblastoma Protein
REV-ERB: Reverse ErbA-related orphan receptor
RORE: Retinoic Acid Receptor-Related Orphan Receptor Response Element
ROS: Reactive Oxygen Species
RTK: Receptor Tyrosine Kinase
SAM: S-Adenosyl Methionine
SASP: Senescence-Associated Secretory Phenotype
SA-β-gal: Senescence-Associated -galactosidase
scRNAseq: Single-Cell RNA Sequencing
SCD: Stearoyl-CoA Desaturase
SIRT1: Sirtuin 1
SOS: Son of Sevenless
SREBPs: Sterol Regulatory Element-Binding Proteins
STING: Stimulator of Interferon Genes
TALL: T cell Acute Lymphoblastic Leukemia
TC: Thyroid Cancer
TCA cycle: Tricarboxylic Acid Cycle (Krebs Cycle)
TGF-β: Transforming Growth Factor Beta
TIS: Therapy-Induced Senescence
TLR: Toll-Like Receptor
TME: Tumor Microenvironment
TNBC: Triple-Negative Breast Cancer
TNF-α: Tumor Necrosis Factor Alpha
Tregs: Regulatory T cells
TSC1/2: Tuberous Sclerosis Complex 1/2
VEGF: Vascular Endothelial Growth Factor
α-KG: Alpha-Ketoglutarate
